# Synthesis and characterization of core–shell magnetic molecularly imprinted polymer nanocomposites for the detection of interleukin-6

**DOI:** 10.1007/s00216-024-05536-x

**Published:** 2024-10-16

**Authors:** Rahil Radfar, Eda Akin, Ekin Sehit, Nastasia Sanda Moldovean-Cioroianu, Niklas Wolff, Rodrigue Marquant, Karsten Haupt, Lorenz Kienle, Zeynep Altintas

**Affiliations:** 1 Bioinspired Materials and Biosensor Technologies, Institute of Materials Science, Faculty of Engineering, Christian-Albrechts-Universität Zu Kiel, Kiel, Germany; 2 Real Structure and Synthesis, Institute of Materials Science, Faculty of Engineering, Christian-Albrechts-Universität Zu Kiel, Kiel, Germany; 3 CNRS Enzyme and Cell Engineering Laboratory, Universite de Technologie de Compiègne, Compiègne, France; 4 Institut Universitaire de France, Compiegne, France; 5 Kiel Nano, Surface and Interface Science (KiNSIS), Kiel University, Kiel, Germany

**Keywords:** Molecularly imprinted polymers (MIPs), Core–shell MIPs, Magnetic nanoparticle functionalization, Biomimetics, Biomarker detection, Interleukin-6 (IL-6)

## Abstract

Interleukin-6 (IL-6) belongs to the cytokine family and plays a vital role in regulating immune response, bone maintenance, body temperature adjustment, and cell growth. The overexpression of IL-6 can indicate various health complications, such as anastomotic leakage, cancer, and chronic diseases. Therefore, the availability of highly sensitive and specific biosensing platforms for IL-6 detection is critical. In this study, for the first time, epitope-mediated IL-6-specific magnetic molecularly imprinted core–shell structures with fluorescent properties were synthesized using a three-step protocol, namely, magnetic nanoparticle functionalization, polymerization, and template removal following thorough optimization studies. The magnetic molecularly imprinted polymers (MMIPs) were characterized using dynamic and electrophoretic light scattering (DLS and ELS), revealing a hydrodynamic size of 169.9 nm and zeta potential of +17.1 mV, while Fourier transform infrared (FTIR) spectroscopy and fluorescence spectroscopy techniques showed characteristic peaks of the polymer and fluorescent tag, respectively. Scanning electron microscopy (SEM) and high-resolution transmission electron microscopy (HRTEM) investigations confirmed the successful encapsulation of the magnetic core within the ca. 5-nm-thick polymeric shell. The MMIP-based electrochemical sensing platform achieved a limit of detection of 0.38 pM within a linear detection range of 0.38–380 pM, indicating high affinity (dissociation constant *K*_D_ = 1.6 pM) for IL-6 protein in 50% diluted serum samples. Moreover, comparative investigations with the non-imprinted control polymer demonstrated an imprinting factor of 4, confirming high selectivity. With multifunctional features, including fluorescence, magnetic properties, and target responsiveness, the synthesized MMIPs hold significant potential for application in various sensor techniques as well as imaging.

**Supplementary Information:**

The online version contains supplementary material available at 10.1007/s00216-024-05536-x.

## Introduction

Cytokines are small proteins with molecular weight in the range of 5–40 kDa that play essential roles in cell signaling pathways to coordinate immune responses and inflammation while influencing cell growth, differentiation, and development. Despite their essential roles in immune responses and physiological processes, however, their excessive and/or unregulated release can have significant detrimental effects [1].

Interleukin-6 (IL-6) is a multifunctional cytokine produced and secreted by a variety of cells, including immune cells like T cells and macrophages, as well as fibroblasts and endothelial cells (non-immune cell types). Discovered in 1986 [2], IL-6 mediates inflammatory and stress responses. Consequently, elevated IL-6 levels can signal cytokine-based pathway activation in acute inflammatory conditions (e.g., sepsis) or chronic diseases (e.g., cancer and/or heart conditions). In inflammatory states, IL-6 levels in body fluids can reach 100 pg mL^−1^ to 1 ng mL^−1^, compared to the normal 5 pg mL^−1^ in serum. Additionally, recent evidence suggests that IL-6 levels are altered after tumor surgery or during chemotherapy, possibly mirroring therapy responses and disease-related factors such as tumor size, recurrence, or stage [3].

It must be noted that elevated IL-6 levels hold crucial significance in the context of postsurgical complications as well [4]. For instance, one of the main complications after colon cancer surgery is anastomotic leakage, which is correlated with elevated levels of IL-6 [5]. Additionally, overexpression of IL-6 can lead to endothelial dysfunction and vascular inflammation, which are risk factors for cardiovascular diseases. IL-6 is also associated with several autoimmune diseases, including rheumatoid arthritis, acute-phase response to infection or injury, and neurodegenerative diseases [6]. Within this framework, it is evident that the advancement of analytical techniques capable of determining IL-6 levels with high precision and sensitivity would be immensely valuable and could represent significant progress in the early diagnosis of pathological conditions such as anastomotic leakage. Hence, researchers in the field of biosensing and medical diagnostics are continuously striving to develop methods that can provide accurate and reliable results for determining IL-6 levels, even in cases where subtle changes are significant indicators of health versus disease.

A variety of natural and synthetic molecular recognition components have been employed in biosensor development for the detection of IL-6, including antibodies [7, 8], IL-6 receptor (IL-6R) [9], aptamers [10], and molecularly imprinted polymers (MIPs) [11–13]. While antibodies, IL-6 receptors, and aptamers serve as valuable recognition elements in biosensing, they do come with certain limitations. On the one hand, antibodies may cross-react with structurally similar molecules (leading to false positives), have limited sensitivity for lower target analyte concentrations, typically require time-consuming and expensive production processes, and are highly sensitive to changes in temperature and pH, hence affecting their stability and shelf life [14]. On the other hand, IL-6R has complex structural features, which can lead to challenges in the engineering of biosensors. Moreover, like antibodies, IL-6 receptors are subject to degradation and denaturation under certain conditions, and their production is time-consuming and costly. Concerning aptamers, they can also interact with various biological components in complex samples (i.e., possessing lower specificity), their selection is a laborious and resource-intensive process, they may degrade over time, and they have limited commercial availability [15]. As a consequence, advanced analytical methods have led to the exploration of innovative materials, such as bioinspired smart materials and MIPs, to improve the detection of target analytes such as IL-6 and the early diagnosis of a wide range of diseases.

MIPs are biomimetic materials with custom-designed molecular binding capabilities. Their synthesis process centers around the complexation of a template molecule, achieved through covalent or non-covalent interactions with functional monomers in a suitable solvent. Subsequently, a three-dimensional polymer network is generated by initiating the polymerization of the monomers in the presence of appropriate cross-linking agents around the template molecules. Once the templates are removed, binding sites are obtained which are characterized by shape, size, and chemical functionality that are well matched to the target molecules [16]. Molecular imprinting has sparked significant interest in the field of analytical chemistry, with applications ranging from solid-phase extraction and liquid chromatography to biosensing, drug delivery platforms, and theranostics [17–19].

With the use of MIPs, a wide range of template molecules can be captured and detected, including viruses [20, 21], bacteria [22], and protein biomarkers [23]. Nonetheless, creating imprints for large molecules poses a significant challenge owing to their substantial size, limited solubility and stability, high structural complexity, and sluggish movement of molecules. An alternative to whole-molecule imprinting is the epitope imprinting technique, where surface-exposed small peptides are identified within the whole target protein and used as molecular templates [24, 25].

MIP nanoparticle synthesis methods vary from bulk polymerization and precipitation methods to suspension, emulsion, and surface imprinting [26]. Among surface imprinting methods, core–shell imprinting holds a prominent position in the design and assembly of various analytical sensors and devices. It is achieved by integrating the MIPs with other functional components, which could constitute either the core or the shell materials. Core–shell molecularly imprinted polymers (CS-MIPs) possess advantageous properties compared to their counterparts due to the presence of their binding sites on the surface of a solid support, resulting in high accessibility to the cavities of MIPs, further simplifying the template removal while enhancing the binding capacity and kinetics. Notably, core materials of inorganic origin, such as silica (SiNP), magnetic nanoparticles (MNPs), quantum dots (QDs) and upconverting phosphors (UCPs), gold nanoparticles (AuNPs), and silver nanoparticles (AgNPs), are commonly used [27]. The main advantage of hybrid inorganic–organic materials lies in their amalgamation of favorable chemical and physical attributes from both organic and inorganic components. In this context, the application of MNPs has garnered significant interest in designing hybrid (organic–inorganic) materials. A particularly valuable aspect is that magnetic molecularly imprinted polymers (MMIPs) combine the magnetic characteristics of MNPs with the remarkable recognition capability of MIPs, resulting in a single hybrid structure possessing multifunctional properties [28].

MMIPs have been used primarily for separation and extraction purposes; however, due to their magnetic characteristics, CS-MIPs can be utilized in applications including magnetic sensing, magnetic resonance imaging, and hyperthermia therapeutics [29]. MMIPs carrying magnetic cores with size over 100 nm simplify the separation and washing steps using an external magnet. Nevertheless, this large size can hinder their application in in vitro and in vivo settings, since particles with larger sizes can lead to toxicity within living organisms. A key advantage of using organic polymeric shells in CS-MIPs is their straightforward functionalization and modification with various functional materials. For instance, conjugation of fluorescent monomers into the polymeric matrix in the MMIP core–shell nanostructures can lead to particles with multifunctional properties, which broadens their applicability beyond that of MMIPs, such as their use in fluorescent imaging.

The employment of MIPs in the detection of IL-6 has been undertaken mainly through electropolymerized MIP films and electrochemical sensors [9, 11, 13, 30–33]. However, Zhong et al. [12] recently developed core–shell MIP structures on the surface of protease–copper phosphate hybrid nanoflowers for the detection and clearance of IL-6 through an enzymatic reaction. Although magnetic MIPs have been used to target a large number of template molecules [34–40], so far, no MMIPs for the detection of IL-6 have been reported. The introduction of MMIPs with specificity for the IL-6 protein as a sample biomarker for inflammatory diseases can open up new opportunities for applications such as biosensing and bioimaging. Therefore, the development of such materials with multifunctional properties and compatibility for use in biological applications would add great value.

In this study, multifunctional core–shell magnetic MIPs with specificity to IL-6 were synthesized for the first time using an epitope-imprinting approach through polymerization of acrylamide-based monomers and a fluorescent monomer in the presence of peptide-immobilized MNPs. In order to synthesize the particles, a series of mathematical calculations and experimental analyses were conducted to establish a synthesis protocol, achieving enhanced properties by optimizing the concentration of the peptide, the reaction media, and the template removal solution. Thorough characterization of the synthesis procedure of MMIPs as well as their post-synthesis properties was carried out using a variety of characterization tools. Dynamic and electrophoretic light scattering (DLS and ELS) techniques were utilized to investigate the size and zeta potential of the particles. Successful synthesis of MMIPs was confirmed using Fourier transform infrared (FTIR) spectroscopy, fluorescence spectroscopy, fluorescence microscopy, contact angle (CA) measurements, scanning electron microscopy (SEM), and high-resolution transmission electron microscopy (HRTEM). The MMIPs were further employed in electrochemical sensor assays to analyze their binding properties. Our work expands the potential applications of MMIPs in early detection of pathologies while also paving the way for prognostic and imaging applications both in vitro and in vivo.

## Experimental section

### Materials and reagents

Citrate-capped magnetic nanoparticle solutions with a concentration of 25 mg mL^−1^ and hydrodynamic size of 50 nm were purchased from Chemicell GmbH (Berlin, Germany). *N*′-Tetramethylethylenediamine (TEMED), 1-ethyl-3-(3-dimethylaminopropyl)carbodiimide (EDC), *N*-hydroxysuccinimide (NHS), phosphate-buffered saline (PBS), *N*-isopropylacrylamide (NIPAm), *N*,*N*′-methylenebisacrylamide (BIS), acrylic acid (AA), *N*-(3-aminopropyl)methacrylamide hydrochloride (APMA), 4-vinylpyridine (VP), ammonium persulfate (APS), bovine serum albumin (BSA), ethanolamine, and sterile human serum were purchased from Sigma-Aldrich (Steinheim, Germany). Acetic acid (AcOH), hexaferrocyanide (K_3_[Fe(CN)_6_]), 2-(*N*-morpholino)ethanesulfonic acid (MES), sodium chloride (NaCl), potassium chloride (KCl), ninhydrin, double-distilled water, methanol (MeOH), and ethanol (EtOH) were purchased from Carl Roth (Karlsruhe, Germany). Carl Roth also provided syringe filters of 0.45 µm pore size. Sodium hydroxide (NaOH) was purchased from VWR (Dresden, Germany). Acryloxyethyl thiocarbamoyl rhodamine B (RhB monomer) was obtained from Polysciences Europe GmbH (Hirschberg an der Bergstrasse, Germany). IL-6, tumor protein p53, cardiac troponin I (cTnI), transferrin, and glucose were all purchased from Sigma-Aldrich (Steinheim, Germany) for sensor studies. All chemicals and solvents were high-performance liquid chromatography (HPLC)/analytical grade and were used without any further purification. PBS solution (10 mM) was used as the buffer unless stated otherwise.

An interleukin-6 epitope peptide was chosen based on its surface accessibility in the IL-6 protein. Using the AlphaFold and Protein Data Bank platforms, the primary sequence of IL-6 protein (PDB: 8QY5) was studied. The N-terminal domain, NH_2_-VPPGEDSKDVAA-COOH, after the signaling part of the protein, showed high surface accessibility and, therefore, was chosen as the epitope. It was synthesized in-house at the CNRS Laboratory for Enzyme and Cell Engineering of Université de Technologie de Compiègne in France, using standard solid-phase-based Fmoc chemistry. Mass spectroscopy and purity analyses of the peptide are shown in Fig. S1.

### Instrumentation and experimental apparatus

During the synthesis process, a mechanical stirrer (Microstar 7.5, IKA-Werke GmbH & Co. KG, Germany) was used for mixing, a centrifuge (Centrifuge 5804 R, Eppendorf Vertrieb Deutschland GmbH, Germany), a vortex mixer (Vortex 2, IKA-Werke GmbH & Co. KG, Germany), and an ultrasonic bath (Bandelin Sonorex Digiplus, Eydam GmbH, Germany) were used during the washing steps for collection and re-dispersion of the magnetic nanoparticles, and a vacuum oven (Vacutherm, Fischer Scientific GmbH, Germany) was employed for drying.

The hydrodynamic size distribution and zeta potential (ZP) of the resulting particles were characterized by DLS and ELS, respectively, using a Zetasizer Pro (Malvern Panalytical Ltd., UK) at a temperature of 25 ± 0.1°C, in backscatter mode. HRTEM (FEI Tecnai F30 G^2^ S-TWIN, USA) and SEM (Zeiss Gemini Ultra 55 Plus, Germany) images were recorded to investigate the morphology of the prepared materials. The SEM images were taken with an in-lens detector to create more contrast and make the details more visible, and the images were analyzed using ImageJ software to obtain the particle size distribution. An FTIR spectrometer (Cary 630, Agilent Technologies, Inc., USA) was fitted with a single-reflection diamond attenuated total reflectance (ATR) module in order to obtain ATR-FTIR spectra in the range of 4000–650 cm^−1^, while an ultraviolet–visible (UV–Vis) spectrophotometer (Cary 60, Agilent Technologies, Inc., USA) was utilized for ultraviolet spectrophotometry to find the optimal concentration of magnetic nanoparticles for further characterization. Contact angle (CA) measurements upon MNP functionalization were performed to study surface wettability changes (Dataphysics OCA 15 Plus with SCA20 software, Dataphysics Instruments GmbH, Germany). MarvinSketch 23.14 software from ChemAxon was used for displaying the peptide structure, conducting elemental analysis of the peptide, measuring the isoelectric point (pI) of the peptide, and calculating the projection areas of ligands.

A fluorescence spectrometer was employed to obtain fluorescence emission spectra of the particles (Cary Eclipse, Agilent Technologies, Inc., USA). Fluorescence microscopy (BZ-X810 coupled with BZ-X800 Analyzer software, Keyence Corporation, Japan) was employed to obtain fluorescent images under ×10 magnification. The binding properties of the MMIPs were studied by voltammetric measurements using a compact electrochemical setup (PalmSens4 Potentiostat with PSTrace 5.9 software, PalmSens B.V., Netherlands) and screen-printed gold electrodes (SPGEs) (counter electrode: Au; reference electrode: Ag; Metrohm DropSens, S.L., Spain).

### Synthesis of MMIPs

**Fig. 1:**
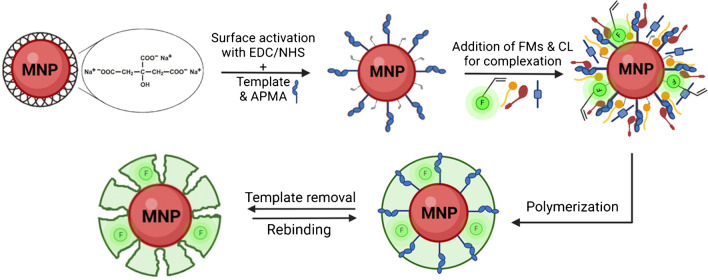
Schematic of the MMIP synthesis process. APMA: *N*-(3-aminopropyl)methacrylamide hydrochloride, F: fluorescent RhB monomer, FM: functional monomers, CL: cross-linker

An epitope imprinting approach was employed to synthesize the MMIPs, facilitating non-covalent interactions including hydrogen bonding, van der Waals forces, and electrostatic interactions between the imprinted binding sites of MMIPs and the target epitope of the IL-6 protein. The synthesis process involved three key stages: (i) functionalization of the MNPs with the epitope template (T) and APMA, (ii) polymerization of the functional monomers (FMs) and cross-linker (CL) around the functionalized MNPs, and (iii) removal of the template molecule from the polymer matrix (TR) (Fig. 1).

#### Functionalization of the MNPs

EDC/NHS-based coupling chemistry was used to covalently attach the peptide from its N-terminal onto the surface of the MNPs, accommodating carboxylic acid groups. The saturation concentrations of EDC/NHS and peptide on the MNPs were mathematically and experimentally investigated to establish the optimized conditions. Three different reaction media, including MES (0.1 M MES, 0.5 M NaCl, pH = 6), water (pH = 6.7–7), and PBS (pH = 7.4), were tested for further optimization.

An aliquot of 400 µL of the MNP stock solution (25 mg mL^−1^) was mixed in 8600 µL of the reaction medium of study, to which 500 µL of EDC solution (40 mM) and 500 µL of NHS solution (50 mM) prepared in the same reaction medium were added. The mixture was maintained under mild stirring using a mechanical stirrer at 100 revolutions per minute (rpm) for 15 min to activate the carboxylic groups of the citrate-capped MNPs. Afterwards, the excess EDC/NHS was removed upon subsequent washing steps by centrifugation. For that, the mixture was centrifuged several times at 10,000 rpm for 10 min, each time taking the supernatant for the next round of centrifugation. This process was repeated until the supernatant was colorless. The colorless supernatant was discarded, and all precipitates were collected in 8000 µL of the same reaction medium of study by quick vortexing, followed by subsequent sonication for 10 min. Then, 1 mL of epitope solution with different concentrations (25–250 µM) and 1 mL of APMA solution (50 µM) prepared in the same reaction medium were added to the MNP solution. The mixture was kept for 2 h at room temperature under mild stirring so that both epitope and APMA could bind to the activated carboxylic groups via their primary amine groups. The particles were collected through multiple rounds of centrifugation and sonication and were finally dispersed in 10 mL of water by 10 min of sonication, and the final solution was stored at +4 °C. Importantly, the purpose of immobilizing APMA on the surface of the MNPs is to ensure that the polymerization occurs at the surface of the particles.

#### Polymerization

**Fig. 2:**
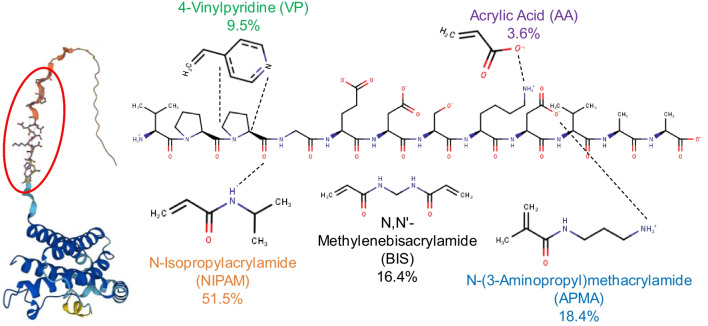
The structure of IL-6 protein from AlphaFold, the accessibility of the epitope chosen in this study, and the molecular structure of the peptide including its possible interactions with different functional monomers

Considering the full coverage of the MNPs with the peptide and APMA, two different polymerization mixtures with different ratios, i.e., T:FM:CL = 1:10:5 (R1) and 1:20:20 (R2), were used for the synthesis of MMIP. The T:FM ratio of 1:10 was chosen based on the minimum number of monomers, considering the potential steric hindrance of the functional groups within the template configuration (i.e., folded loop, as shown in Fig. S2), which would result in a first layer of polymeric shell in the case that all the monomers were completely polymerized. For this batch, half of the functional monomer ratio was considered for the cross-linker (5) to gain moderate cross-linking. The FMs and their ratios in the polymerization mixture were determined considering the functional groups on the template and the monomers that induce various non-covalent interactions (Fig. 2).

NIPAm (51.5 mol%) was chosen for hydrogen bond formation with the C=O groups on the template molecule, APMA (18.4 mol%) and acrylic acid (3.6 mol%) were chosen for their electrostatic interactions with the acidic and basic groups on the peptide, respectively, and VP (9.5 mol%) was chosen for C–H/π and spatial interactions with the ring in prolines [41]. BIS (16.4 mol%) and water were used as the cross-linking agent and solvent, respectively [42]. RhB monomer (0.6 mol% of the total polymerization mixture) was employed as the fluorescent tag for further investigation of the polymer matrix stability through their emission properties. The total concentration of FMs and CL in solution was 30% (w/v), while the concentration of the APS initiator was set as 0.5% (w/v), both guided by prior findings [43].

In order to prepare the R1 polymerization solution, 7.1 mg of NIPAm, 4 mg of APMA, and 3.08 mg of BIS were dissolved in 41 mL of water in a 100 mL round-bottom flask (RBF), to which 1 mL of RhB monomer solution (0.5 mg mL^−1^ in EtOH) was added. Separately, 0.3 µL of AA and 1.24 µL of VP were dissolved in 2 mL of water, and the solutions were added to the previous monomer solution. Then, 5 mL of the as-prepared peptide-immobilized MNP solution was added to the monomer mixture. The RBF was then capped and sealed with a rubber septum and was degassed under simultaneous vacuum and sonication for 15 min. Afterwards, it was purged with nitrogen gas for 30 min. In a separate beaker, 1 mg of APS was dissolved in 20 mL of water, after which 10 µL of this solution and 30 µL of TEMED were mixed in 960 µL of water as the initiator solution, which was added to the monomer mixture under nitrogen atmosphere and mild stirring to start the polymerization. TEMED was used as a catalyst to initiate APS at room temperature. After 2 h, the polymerization was quenched by uncapping the RBF and exposing the polymer solution to air.

In the R2 polymerization solution, the amount of each functional monomer was doubled, while the cross-linker was four times higher. The other conditions were kept constant.

#### Template removal

Different TR solutions, including a mixture of MeOH:AAc (9:1) (100%, 75%, 50%, and 25%), ninhydrin solution (2%) in ethanol, and NaOH solution (0.1 M and 0.05 M), were examined for the TR step. To remove the template, the polymerization mixture with MMIP solution from the previous step was initially centrifuged multiple times at 10,000 rpm for 10 min, each time taking the supernatant for the next round until the supernatant did not show any shades of orange coming from the bare MNPs. Afterwards, all the precipitates were collected by 5 mL of the TR solution and sonicated at 35–40 °C for 10 min. To wash the particles after the TR, they were treated twice with 5 mL of water by centrifugation and sonication. Finally, the particles were collected in 5 mL of water to obtain a concentration of 1 mg mL^−1^ and were stored at +4 °C for further characterization.

### Electrochemical studies

The binding behavior of MMIPs towards the epitope and IL-6 was studied using the square-wave voltammetry (SWV) technique (Table S1) in buffer and serum. Prior to detection, 10 µL of the as-synthesized MMIPs (0.05 mg mL^−1^) was drop-cast on the gold surface of the SPGEs overnight for MMIP immobilization through adsorption. The first signal was recorded in the presence of a redox marker solution (10 mM K_3_[Fe(CN)]_6_ with 0.1 M KCl in water) after incubating the MMIP-screen-printed electrode (SPE) with PBS. Then, the MIP-free areas of the sensing surface were blocked by incubating the MMIP-SPE with BSA solution (5 mg mL^−1^ in PBS) and further ethanolamine solution (1 mM in PBS, pH: 8.5) for 5 min each. Between each incubation, the surface was washed once using PBS. Then, the reference signal was recorded using the redox marker solution after incubating the MMIP-SPE with PBS for 15 min. Afterwards, different concentrations of analyte solutions (either in PBS or 50% diluted human serum in PBS) were successively incubated for 15 min starting from the lowest concentration. After incubation with each concentration, the surface was washed three times with PBS, and the signal readout was conducted using the redox solution. The amount of the solutions for incubations, washing, or signal readout was 40 µL. Voltammetry readouts were recorded in triplicate, and the average was used as the signal. Finally, the relative signal with respect to the reference was calculated based on the literature [44] as the sensor response.

## Results

### Optimization studies

In the carbodiimide-based coupling approach, the concentration of the cross-linking agents (EDC/NHS), the immobilized moiety (i.e., the epitope peptide), and the reaction media are critical to obtaining successful conjugation [45–47]. Therefore, saturation concentrations of EDC/NHS and the peptide on the MNPs were calculated using a mathematical approach (Section 1 in Supporting Information) to find the maximum coverage of carboxylic groups and peptide on the surface area of a spherical particle with a diameter of 50 nm, based on the projection areas of the ligands (Fig. S3a). The results showed that for each mole of magnetic nanoparticles, 40,000 mol EDC/NHS and 4000 mol peptide are required. Experimental trials were conducted to confirm our calculations. Titration experiments are usually used for finding the saturation concentration of a linker [48]; however, in the case of MNPs, these experiments could not provide a reliable signal, since the UV–Vis spectra of the magnetic particles overlaid the hydrolysis peak of EDC/NHS at 260 nm (Fig. S3b, c). Therefore, double the mathematically calculated amount of EDC/NHS was chosen based on the literature [47] in order to activate the carboxylic groups on the citrate-capped magnetic nanoparticles.

The optimal peptide concentration was studied using DLS characterization (Fig. S4a, b). The hydrodynamic size of the magnetic nanoparticles increased as the peptide concentration increased up to 100 µM, after which it remained constant despite the increased peptide concentration. Therefore, the 100 µM peptide concentration was considered as the saturation point, which agreed with our mathematical calculations. APMA monomer was anchored on the MNPs during the template immobilization stage to ensure the coverage of the polymer shell around the core. Thus, half of the saturation concentration was used for immobilization, while the other half was allocated to APMA conjugation.

DLS and ELS characterization methods were employed to analyze the immobilization of template peptide in different media (Fig. S4c, d). The functionalization of the MNPs in water and PBS resulted in increased hydrodynamic size of the peptide-immobilized particles, while no alteration was observed when MES was used, suggesting that the immobilization was not successful in MES buffer. This could be due to the relative acidic pH value of the MES solvent providing H^+^ ions in the media, which can screen the negative ionic state of the carboxylic groups. Conversely, the ZP values of particles after immobilization determined the optimal solvent between PBS and water. As shown in Fig. S4d, the change in the ZP of particles treated in water was greater than that in PBS. This resulted in lower stability of the particles in water after functionalization, and higher rates of agglomeration. Thus, PBS was identified as the optimal media of reaction for peptide immobilization on the MNPs.

DLS, ELS, FTIR, and fluorescence spectroscopy were employed to determine the optimal MMIP fabrication conditions. A mixture of MeOH:AAc = 9:1 was chosen based on the literature [49] (${TR}_{MeOH:AAc/100{\%}}$) to remove the covalently bound peptide after polymerization (T:FM:CL = 1:10:5). However, upon TR, a clear pinkish color in the supernatant was visible, suggesting that the polymer with RhB was removed (Fig. S5a). To investigate this assumption chemically, FTIR spectra of the particles after each step of synthesis and the peptide were studied and compared (Fig. S5b). The characteristic FTIR peaks of the IL-6 peptide were at three main regions, including 3400–2700 cm^−1^, 1800–1300 cm^−1^, and 1300–900 cm^−1^. In the upper wavenumber region (3400–2700 cm^−1^), the peaks attributed to the N–H stretching were from primary and secondary amine groups in the peptide structure, O–H stretching of intramolecular hydrogen bonding including the −OH in carboxylic groups [50], and finally the C–H stretching of the alkane groups, from the higher wavenumber to the lower, respectively. In the middle region, peaks—starting from higher wavenumbers—were related to the C=O stretching of primary amide groups and carboxylates [51]. Also, C–H bending of alkane and/or O–H bending of carboxylates or alcohol were observed. In the lower wavenumber region, C–N and C–O stretching of amine groups or esters and C–O stretching of primary alcohol groups were visible [52, 53].

Moreover, the characteristic bands of MNPs comprised one broad peak in the region of 3500–3000 cm^−1^ and two relatively strong and sharp peaks between 1750 and 1250 cm^−1^. These peaks corresponded to the O–H stretching from carboxylates, intermolecular hydrogen bonding between the surface of MNPs and the citrate groups, C=O stretching of carboxyl groups, and O–H bending of carboxylates, respectively. In the case of peptide-immobilized MNPs, the peptide peaks in the upper and medium regions were superimposed by the peaks of MNPs. However, the peak in the lower wavenumber region (at 1000 cm^−1^) confirmed successful immobilization of peptide on the surface of MNPs. Upon polymerization, the new peak in the region of 3000–2750 cm^−1^ was attributed to the C–H stretching of alkane groups. Also, after polymerization, the peptide peak at the lower wavenumber region was preserved [52, 53].

The primary results obtained from FTIR after ${TR}_{MeOH:AAc/100{\%}}$ confirmed the hypothesis of polymer removal upon TR, since in addition to the weaker polymer peaks, the characteristic bands of MNPs ascribed to the –OH stretching in carboxylic groups were also weaker in the FTIR spectra of the sample after ${TR}_{MeOH:AAc/100{\%}}$. Therefore, lower concentrations (25%, 50%, and 75%) of MeOH:AAc mixed with water for the treatment of the particles were also tested. FTIR, DLS, and ELS characterizations (Fig. S6) suggested that 50% concentration might have preserved the polymer and can be used for further characterizations. To confirm this, fluorescence spectroscopy was conducted (Fig. S7), which did not show characteristic behavior of RhB fluorophore, indicating the removal of the polymer.

One reason for this could be the insufficient amount of functional monomer and cross-linking agents in the polymerization solution. It is worth mentioning that APMA is immobilized on the surface of the MNPs the same way as peptide, with the hypothesis that the large number of covalent bonds between the monomers, and stability of the polymer matrix due to the cross-linking effect would preserve the polymer structure. However, if the polymer coverage and cross-linking are not sufficient, instead of achieving a fully cross-linked matrix covering the whole MNPs, it would be highly possible that some polymeric matrix would form only around the template molecules bound to the MNPs through APMA (Fig. S8). To mitigate this risk, the ratio of the polymerization mixture was altered by T:FM:CL = 1:20:20.

In addition, it should be taken into consideration that the pI of peptide plays a significant role in peptide isolation and separation, which is crucial in the TR step in epitope imprinting. Therefore, the pI of the peptide was calculated using MarvinSketch software and was determined to be 3.75, as can be seen in Fig. S9. This is indicative of the fact that in TR solutions with higher pH, the peptide becomes more negatively charged, facilitating higher solubility. Therefore, alternative TR solutions, including 50% MeOH:AAc (pH = 3), 2% ninhydrin solution (pH = 5), and 0.1 M and 0.05 M NaOH solution (pH = 12.7 and 13, respectively), were tested. Of note, ninhydrin was chosen due to its chemical reaction with proline residues, which could break the epitope from the proline sites. The results obtained from fluorescence spectroscopy and imaging, in addition to those of DLS, ELS, and FTIR (Figs. S10, S11), showed that the R2 ratio yielded enhanced stability of the polymeric shell, while the best TR solution, with respect to preserving the polymer shell by showing a discrete and high fluorescence emission intensity, was 0.05 M NaOH, which was utilized for TR in the remainder of the study. The empirical conclusion that the 0.05 M NaOH solution was the most suitable TR solution confirmed the importance of the pH in the TR step with regard to the pI of the peptide.

### Characterization of the MMIPs

**Fig. 3:**
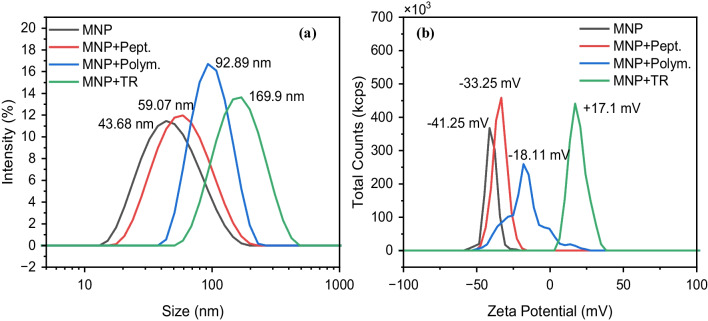
Hydrodynamic size distribution (**a**) and zeta potential (**b**) of bare magnetic nanoparticles (MNP), peptide-immobilized magnetic nanoparticles (MNP+Pept.), after polymerization (MNP+Polym.), and after TR with 0.05M NaOH (MNP+TR.). Measurements were performed in water

The hydrodynamic size and charge of the MNPs upon peptide immobilization, polymerization, and TR at optimized conditions were studied using DLS and ZP analysis (Fig. 3). As shown in Fig. 3a, upon peptide immobilization, the size of the particles increased slightly (ca. 15 nm), while the increase in the size was more prominent upon polymerization (ca. 34 nm) and TR (ca. 77 nm). It should be noted that centrifugation also played a role in the size increase; however, the variation in size observed in this instance cannot be attributed entirely to centrifugation, since samples with different peptide concentrations at similar centrifugation conditions possessed different hydrodynamic sizes (Fig. S3a, b). The increase in the size upon TR was associated with agglomeration or swelling effects of the polymer layer after treatment with TR solution.

The presence of different species, i.e., peptide, APMA, and templated and template-free polymer shell, on the surface of the MNPs was also confirmed by the ZP results (Fig. 3b). As expected, the ZP of the bare magnetic particles was negative (−41.25 mV), which was due to the presence of carboxylic acid groups of the citrate coating. When peptide and APMA were immobilized on the MNP surface, the ZP decreased to −33.25 mV. This can be attributed to the fact that, upon immobilization, the carboxylic acid groups form amide bonds with the peptide or APMA, making the surface less negatively charged. On the other hand, after polymerization and template removal, the ZP increases again (−18.11 mV and +17.1 mV, respectively), which clearly suggests changes in the surface properties after each step.

**Fig. 4:**
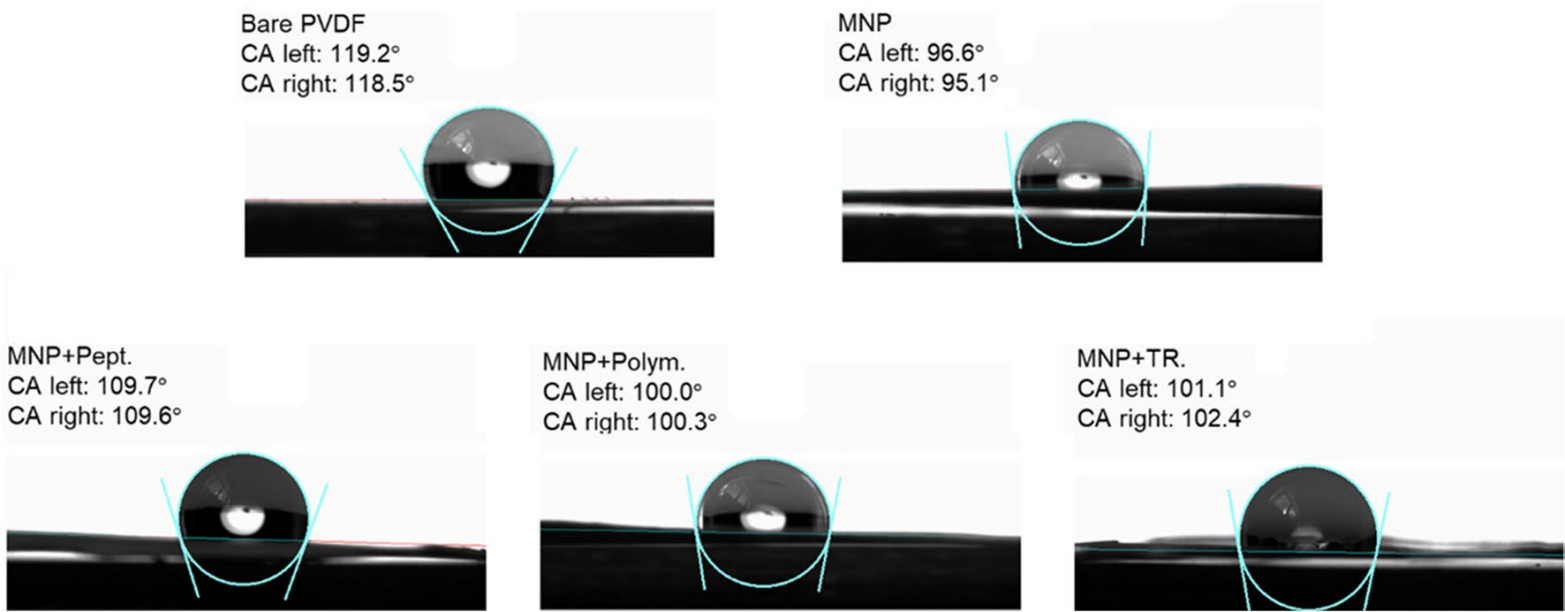
The contact angle measurements of bare PVDF membrane, PVDF membrane after being coated with MNPs, peptide-immobilized MNPs, and MNPs after polymerization and TR with 0.05 M NaOH

Contact angle (CA) measurements were performed to investigate the wetting behavior of MMIPs after each step of functionalization to ensure successful modification of the MMIP surface (Fig. 4). The samples for CA measurements were prepared on polyvinylidene difluoride (PVDF) membranes as the solid surface, which was drop-coated with a mixture of ethanol and the sample solution (1:1 volume ratio). Ethanol was incorporated into the sample solution to improve the sample coverage on hydrophobic PVDF substrate (ca. 118.85 $^\circ$). The CA value decreased after MNP coating on the substrate (ca. 95.85 $^\circ$) since MNPs are hydrophilic due to the carboxylic acid groups of the citrate-capped MNPs. On the other hand, upon peptide immobilization, the CA increased to 109.67°, indicating enhanced hydrophobicity of the peptide-immobilized particles, as the template peptide accommodates hydrophobic residues such as proline, valine, and alanine. As expected, upon polymerization, the CA decreased to 100.15°, since the polymer layer provides more hydrophilicity due to the amine groups. No significant change in CA was observed upon TR (101.75°), since the polymer is still the major surface component in determining the CA.

**Fig. 5:**
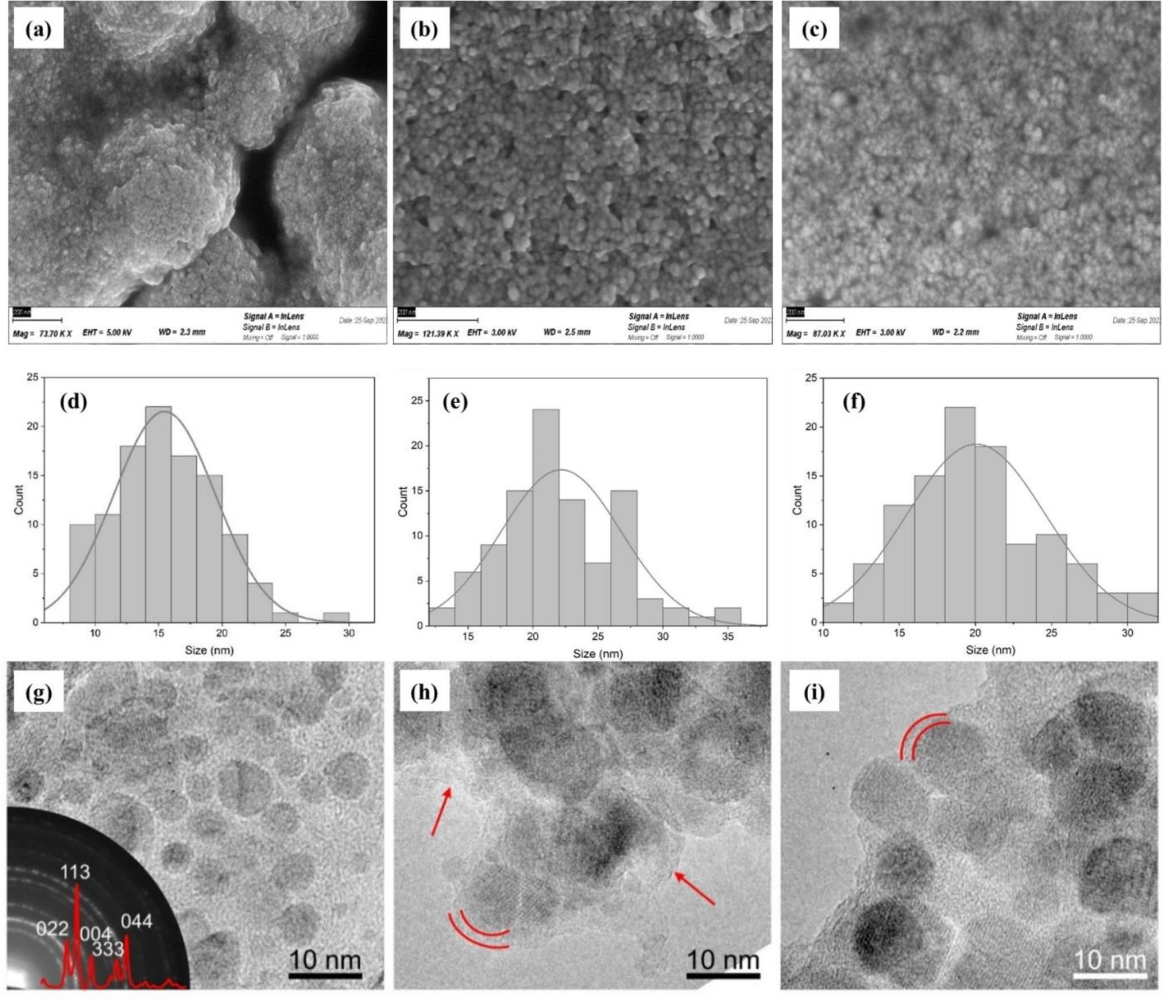
SEM images of **a** untreated MNPs, and MNPs after **b** polymerization and **c** TR with 0.05 M NaOH. **d−f** The corresponding size distributions obtained from SEM images. HRTEM images of **g** bare MNPs, and MNPs after **h** polymerization and **i** TR with 0.05 M NaOH

The particle morphology after polymerization and TR was studied using SEM and HRTEM (Fig. 5). For SEM imaging, samples were dried in a vacuum oven at 45 $^\circ{\rm C}$ overnight, while for HRTEM imaging, solutions of 0.03 mg mL^−1^ for each sample were prepared and drop-coated on the carbon–lacey Cu TEM grids. Size distribution analysis of the SEM micrographs (Fig. 5d–f) revealed smaller sizes (ca. 15 nm for bare MNPs) in comparison to the hydrodynamic sizes recorded with DLS (ca. 44 nm for bare MNPs), as expected. However, the trend in the size increase upon different modification steps, as shown by the SEM images, was similar to the one observed in DLS measurements. The average diameters for bare MNPs, polymer-coated MNPs, and MMIPs were measured as 15 nm, 22 nm, and 20 nm, respectively. It is worth noting that drying the particles in a vacuum oven causes them to adhere to one another due to their magnetic properties, leading to significant agglomeration, as also illustrated in the SEM images (Fig. 5a–c).

HRTEM images (Fig. 5h and i) showed the formation of an amorphous layer, most likely the polymeric shell coated around the particles. Of note, the bare (Fig. 5g) and coated MNPs were dispersed on TEM grids with an amorphous holey carbon support layer, adding a diffuse contrast between the particles. To circumvent this issue, for the inspection of the MNP after polymerization and TR with 0.05 M NaOH, sample areas at the edge of the carbon film were taken to ensure that the diffuse contrast between nanoparticle agglomerations stemmed from the polymeric shell. The bare magnetic nanoparticles show a size distribution in the range of 5–15 nm, confirming the SEM results. Selected-area electron diffraction was also performed on the MNPs, confirming the ${\text{Fe}}_{3}{{\text{O}}}_{4}$ (magnetite, *Fd*3*m*) phase. The size distribution of the imaged particles after polymerization and TR is larger than that of the bare MNPs, which stems from the effect of centrifugation during sample preparation. Following the TR treatment, the polymeric shell was observed in a less condensed manner, with a thickness of 1–5 nm (*n* = 10), suggesting its partial removal.

**Fig. 6:**
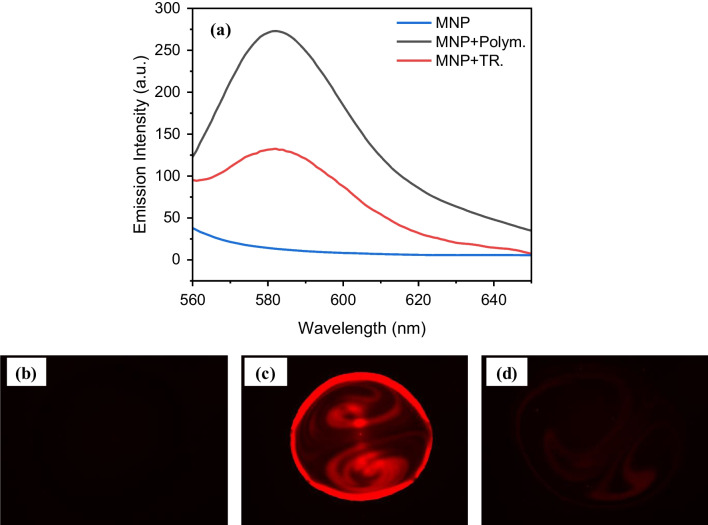
**a** Fluorescence spectra of MNPs, and MNP after polymerization and TR with 0.05 M NaOH, excited at 530 nm with an excitation slit of 5 nm, emission slit of 20 nm, and a photomultiplier tube voltage (PMT) of 800 V. Fluorescence microscopy images of **b** bare MNP, **c** MNP after polymerization, and **d** MNP after TR with 0.05 M NaOH in monochromatic red mode

To study the effect of the TR with 0.05 M NaOH on the polymer matrix, fluorescence spectroscopy was conducted to evaluate the alteration of the fluorescence emission (λ_exc_ = 530 nm) of the RhB-incorporated MMIPs before and after TR (Fig. 6a). The characteristic emission peak of RhB recorded at 580 nm was reduced following the TR. It is likely that the ester or thiocarbamate functions in the link with RhB were hydrolyzed by the NaOH treatment, leading to reduced emission intensity. Moreover, fluorescence microscopy was employed as a complementary characterization tool to visualize the fluorescent properties of the particles after the TR, confirming the results of the fluorescence spectroscopy. For this, one droplet (10 $\mu L$) of sample was dripped on the glass slide, and images were captured in the red monochromatic mode with 100% brightness and 0.3 s exposure time using a ×2 lens. The reduced emission of the sample after TR was in agreement with the fluorescence spectroscopy investigation (Fig. 6b–d).

All the obtained results confirmed the successful synthesis of our multifunctional MMIPs with fluorescent properties.

### Electrochemical detection

#### Affinity and sensitivity studies in buffer

**Fig. 7:**
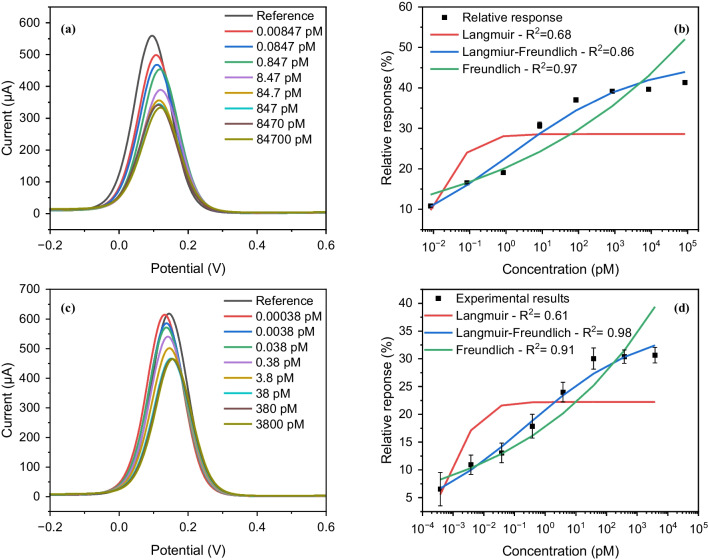
Square-wave voltammograms of binding experiments to IL-6 epitope (**a**) and protein (**c**) with seven different concentrations in PBS, and overall results of concentration-dependent assays using SWV (*n* = 3) fitted to Langmuir, Freundlich, and Langmuir–Freundlich adsorption models for both epitope (**b**) and protein (**d**)

The binding properties of the as-synthesized particles were studied using electrochemical characterization methods. SWV was utilized to investigate the binding behavior of MMIPs towards the epitope (Fig. 7a, b) and to the whole IL-6 protein (Fig. 7c, d). The MMIP-coated SPGE sensing platform was exposed to varying concentrations of peptide (from 0.01 pg mL^−1^ to 100,000 pg mL^−1^, i.e., 0.00847 to 84,700 pM, with peptide molecular weight of 1180.55 g mol^−1^) in buffer to confirm the affinity of the MMIPs towards its template. Following the sample incubation on the MMIP-SPE platform, the voltammogram was recorded in the presence of the redox solution. The highest current value was obtained for the reference measurement in which the MMIP modified sensor was treated with a buffer solution without the analyte. After incubating each concentration of the analyte molecule for 15 min, the surface of the SPE was gently washed once with PBS, and then the current signal was recorded three times using the redox marker. The relative signal with respect to the reference was calculated using the average current after each incubation. As the sample solution with peptide was introduced to the sensing surface, the peptide molecules bound to the cavities of the MMIPs. The occupation of cavities resulted in hindered diffusion of the redox marker onto the gold electrode, leading to decreased current readout. Therefore, by increasing the concentration of the target, the current signal was further suppressed with a linear proportionality to the concentration of the analyte. However, at a later point, the sensor reached saturation, i.e., loss of linear regime, since all cavities of the MMIPs were occupied, and no more molecules could bind to the surface.

In the case of epitope binding experiments, the concentration-dependent relative signal plot showed a saturation point at higher concentration values starting from 840 pM (i.e., 1000 pg mL^−1^), leading to a linear range of 0.0084–840 pM, with an experimental limit of detection (LOD) of 0.0084 pM. The binding isotherm was fitted to three different models, including Langmuir, Freundlich, and Langmuir–Freundlich isotherms. Only the Langmuir–Freundlich model yielded an acceptable fit, with *R*^2^ = 0.97, revealing a dissociation constant (*K*_D_) of 1.29 pM. The Langmuir binding model assumes homogeneous binding sites, while the Freundlich model considers the heterogeneity of the binding sites [44, 54]. The hybrid Langmuir–Freundlich model combines both Langmuir and Freundlich binding trends to explain the binding behavior of MIPs in saturation and sub-saturation concentrations [54] and takes into account the nonspecific binding above a certain concentration.

Once the successful binding of MMIPs with the epitope was confirmed, the electrochemical detection of IL-6 protein was investigated. The sensor assay developed for the detection of IL-6 in buffer with a concentration range of 0.00038–3800 pM (i.e., 0.01–100000 pg mL^−1^ with IL-6 molecular weight of 26 kDa) showed saturation at concentrations above 38 pM (i.e., 1000 pg mL^−1^), leading to a linear range of 0.00038–38 pM. Similar to the epitope binding assay, the binding isotherm for IL-6 also demonstrated Langmuir–Freundlich-based binding behavior, which may be attributed to the existence of some degree of heterogeneity in the binding sites of cavities. In this case, the sensor revealed a *K*_D_ of 0.25 pM and an LOD of 0.00038 pM (i.e., 0.01 pg mL^−1^).

#### Selectivity and sensitivity studies in buffer

**Fig. 8:**
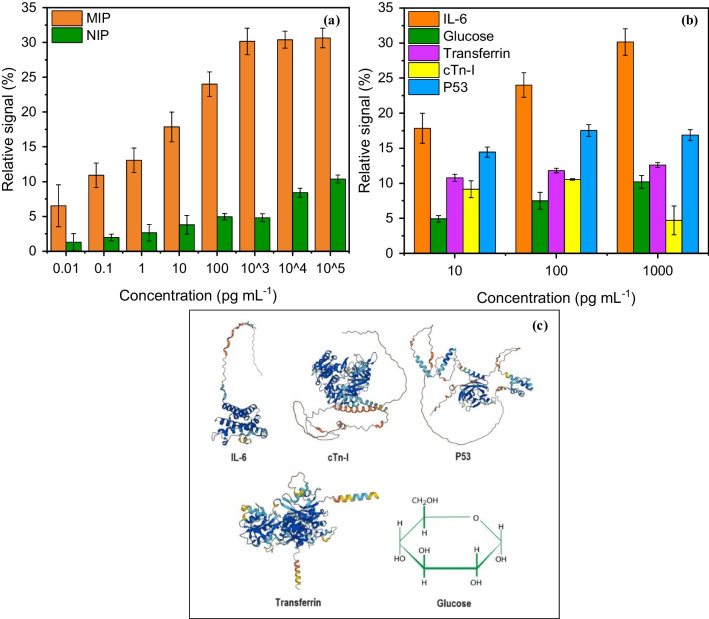
**a** Selectivity studies and **b** specificity studies of MMIP, along with **c** the representation of the secondary structure of the cross-reactant protein species from the AlphaFold database and the chemical structure of glucose

The selectivity of the MMIPs was examined in comparison to the response obtained from magnetic non-imprinted polymers (MNIPs). MNIPs were synthesized and treated the same as MMIPs but in the absence of the template molecule. As shown in Fig. 8a, the absence of specific cavities on the MNIPs caused lower responses. The imprinting factor was calculated by averaging the response from the MMIPs over the average response from MNIPs, yielding a value of 4.28.

Specificity studies were conducted by investigating the response of the MMIP sensor to different concentrations of cross-reactant molecules, including transferrin, cTnI protein, p53 protein, and glucose (Fig. 8b). MMIPs showed the highest signal for the IL-6 protein, confirming their preferential specificity. However, the sensor also demonstrated considerable cross-reactivity towards proteinaceous interferents, namely, p53 and transferrin. This moderate specificity of the synthesized MMIPs might be attributed to several factors, including the presence of similar amino acid sequences as well as the shape resemblance of the other cross-reactants to IL-6. To better understand the slight lack of specificity, studies with human serum albumin or computational simulations can be performed, which are planned for the future.

#### Serum studies

**Fig. 9:**
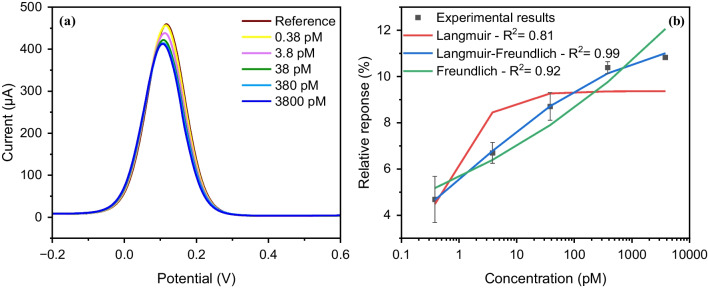
**a** Square-wave voltammograms of IL-6 protein binding with five different concentrations in 50% human serum, and **b** overall results of concentration-dependent protein binding assay using SWV (*n* = 3) fitted to Langmuir, Freundlich, and Langmuir–Freundlich adsorption models

The MMIP sensor was also employed for the detection of IL-6 protein in human serum to evaluate its applicability in complex media. The reference signal was recorded after incubating the MMIP-SPE sensor with 50% diluted serum with PBS without IL-6 spiking for 15 min. Afterward, different concentrations of IL-6 (0.38–3800 pM) in 50% human serum were applied on the sensing platform for 15 min prior to the SWV measurement with the redox marker. As expected, the diluted human serum significantly suppressed the current signal since it contains a large variety of biomolecules that can nonspecifically bind to the sensor surface (Fig. 9). Despite the current suppression by the serum, however, increasing the concentration of IL-6 further reduced the signal, reflecting sensitive binding even in complex medium. No significant response was recorded at concentrations lower than 0.38 pM, resulting in an experimentally determined LOD of 0.38 pM (i.e., 10 pg mL^−1^). Saturation was observed at concentrations above 380 pM, leading to a linear detection range of 0.38–380 pM. The binding isotherm was fitted to the three models as mentioned above, with the Langmuir–Freundlich model having the best fit and the highest *R*^2^ value, resulting in a dissociation constant of 1.6 pM. The high sensitivity and affinity for IL-6 in serum samples show the potential for use of MMIP-SPE in real-life applications.

#### Statistical analysis

In order to better understand the relation between the data obtained from the binding, selectivity, and specificity studies, one-way statistical analysis was employed using analysis of variance (ANOVA) with an in-house script. This statistical analysis considers the null hypothesis when there are no statistically significant variances. When high *F*-statistics, and as a result low *p*-values, are calculated using the analysis, the null hypothesis should be rejected, since they denote significant differences in the studied groups. Four different groups of data were compared including the binding of MIPs to the peptide versus protein in buffer, IL-6 binding to MMIPs in buffer and serum, IL-6 binding to MMIPs versus MNIPs, and finally, the binding of MMIPs to different analyte molecules as in cross-reactivity tests. The *F*-statistic and *p*-value for each group are listed in Table 1.

**Table 1:** *F*-statistics and *p*-values obtained for different groups of study in statistical analysis

	*F*-statistics	*p*-value
Peptide vs. protein	17.49	0.00027
Buffer vs. serum	0.79	0.39
MMIPs vs. MNIPs	12.99	0.0012
IL-6 vs. other cross-reactants	12.10	0.00076

**Fig. 10:**
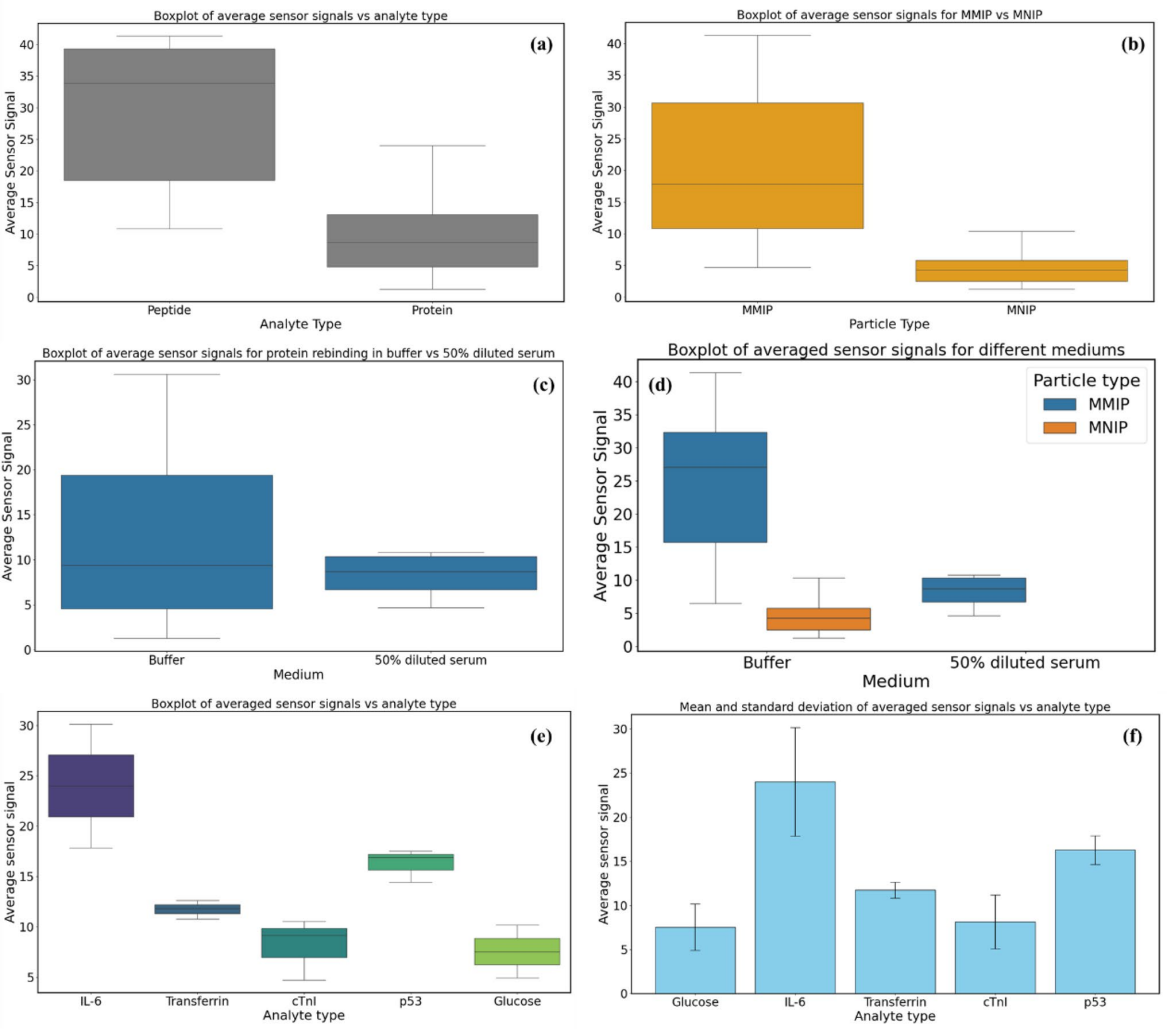
Statistical analysis of the sensor data. **a** Comparison of peptide and protein binding in buffer, **b** comparison of MMIP and MNIP sensing results, **c** analysis of the sensor signals in different media (buffer vs. serum), **d** overall analysis of the IL-6 binding results in buffer and serum to MMIPs and MNIPs, *e* analysis of the data obtained from cross-reactivity studies

Significant differences between the sensor signals for peptide and protein analytes were observed (Fig. 10a–d), with peptide showing higher sensor signals, when compared to IL-6 whole cytokine. Also, significant variance was obtained between these two, leading to high *F*-statistics (17.49) and low *p*-value (0.00027), indicating higher sensitivity of MMIP for the IL-6 peptide. This was expected, since through epitope imprinting, the cavities are mainly complementary to the epitope itself and only bind to the IL-6 through the peptide region. In the other group, MMIPs significantly outperform MNIPs, with higher sensor signals and broader ranges, revealing the outstanding role that the binding cavities on the MMIPs played in capturing IL-6 specifically. On the other hand, the medium in which the analyte was dispersed, i.e., buffer versus serum, did not play a significant role in variance, having a very low *F*-statistic. Due to the complexity of the serum medium comprising many different macromolecules, the sensor signals in the case of serum were lower, while buffer enhanced the sensor signals due to a reduced number of interfering molecules. However, the sensor signals from MMIPs in serum were still higher than those of MNIPs in buffer. Overall, MMIPs in buffer showed the highest signals, with a wide range distribution, followed by MMIPs in diluted serum, with reduced but consistent signals (Fig. 10d).

Moreover, the statistical analysis of the data obtained from cross-reactivity experiments (Fig. 10e), with different analyte types as the independent group and averaged sensor signals as the dependent group, resulted in an *F*-statistic of 12.11 and a *p*-value of 0.00076, indicating large differences between the group averages relative to the variability within the groups in addition to statistically significant differences between the analyte types. IL-6 showed the highest median sensor signal and the widest range of values (i.e., significant variability). In contrast, transferrin and cTnI had relatively narrow ranges and lower median values coming from weaker sensor responses. While p53 possessed a moderate range and higher median sensor signal compared to transferrin and cTnI (i.e., stronger responses but still less than IL-6), glucose had the lowest median and a narrow range. Tukey’s honestly significant difference (HSD) was applied to assess the significance of differences through multiple comparisons of means between IL-6 and other cross-reactants (Fig. 10f), which further confirmed that IL-6 had the highest variations and was significantly different from the other cross-reactants. These results indicate that binding to IL-6 is statistically higher, illustrating the specificity of the MMIPs toward IL-6 in comparison to the other analytes.

#### Stability studies

**Fig. 11:**
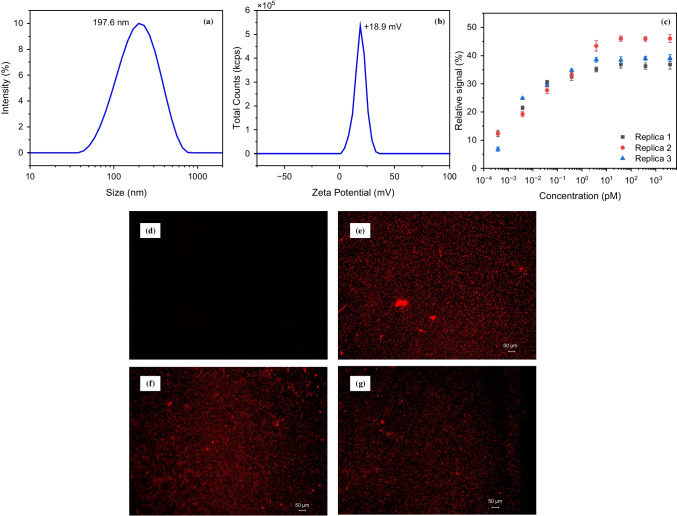
**a** DLS and **b** ELS of MMIPs stored at +4 °C for 6 months. **c** Protein binding assays of three different sensor assays using the MMIPs stored at +4 °C for 6 months. Fluorescent images (×10 with exposure time of 1.6 s) of the electrochemical sensor chip **d** before drop casting the MMIPs overnight, **e** 1 day and **f** 6 months after drop casting the MMIPs, and **g** after binding experiments were conducted on the chip which was kept for 6 months after the immobilization of the MMIPs

In order to investigate the stability of the MMIPs, DLS and ELS characterization was conducted on the particles after 6 months of storage at +4 °C to evaluate the change in their hydrodynamic size and ZP. The DLS results showed a unimodal size distribution with a peak size of 196.77 nm (Fig. 11a), indicating a slight increase in the hydrodynamic size, which may be attributed to the agglomeration of MMIPs and swelling of the polymeric layer. In addition, ELS resulted in a positive ZP of +18.9 mV, similar to the particles directly after synthesis with a ZP of +17.1 mV (Fig. 11b). Furthermore, three different protein binding assays were performed in buffer using MMIPs which were stored for 6 months at +4 °C. All three sensors showed the same binding trend as previous studies, with a linear detection range of 0.01–100 pg mL^−1^, an LOD of 0.01 pg mL^−1^, and a saturation point above 100 pg mL^−1^, leading to reproducible results (Fig. 11c).

To investigate the stability of the sensing platform, fluorescent images of the sensor electrode were acquired before and after 6 months of storage and protein binding assay (Fig. 11d–g). The sensing platform used in these experiments was prepared by drop casting MMIPs on the SPEs 6 months before the time of the experiment. Since MMIPs possessed fluorescent properties due to the presence of RhB monomer in the polymerization mixture, their immobilization on the SPEs was confirmed using fluorescence microscopy (Fig. 11d and e). Not only were the particles immobilized successfully on the chip surface, but they also remained on the chip over 6 months (Fig. 11f). Moreover, after the binding experiments, which included several washing steps, the presence of the MMIPs on the surface was clearly visible (Fig. 11g).

## Discussion

The results obtained in this study confirm the successful synthesis of our multifunctional core–shell MMIPs designed for detecting IL-6. The MMIPs were employed in a label-free electrochemical sensor through direct detection over 15 min, exhibiting competitive affinity (0.25 pM in buffer and 1.6 pM in serum), sensitivity (LOD of 0.00038 pM [i.e., 0.01 pg mL^−1^] in buffer, and 0.38 pM [i.e., 10 pg mL^−1^] in serum), and selectivity (imprinting factor [IF] = 4.28), surpassing existing reports [55–58]. Zhang et al. reported an amperometric electrochemical immunosensor by employing capture antibody-conjugated magnetic nanobeads, while horseradish peroxidase (HRP) and detector antibodies aided sandwich assay-based detection. After immobilizing the capture antibodies on the working electrode using a magnet, different concentrations of IL-6 were incubated overnight prior to detection, leading to an LOD of 0.42 pg mL^−1^ in buffer [55]. In another work, Tan et al. developed a voltammetric electrochemical sensor platform for label-free detection of IL-6 using graphitic carbon electrodes which were prepared by direct laser scribing of polyimide tapes and modified with capture antibodies. Before performing the signal readout using a redox marker, different concentrations of IL-6 were incubated for 90 min, resulting in an LOD of 5.1 pg mL^−1^ in PBS [56].

In contrast, Tertis et al. employed aptamers for quantitative detection of IL-6 in electrochemical sensors. To immobilize the thiolated aptamers on the surface of the electrodes, several modification steps were performed on the glassy carbon electrodes (GCEs), including functionalization of GCEs with *p*-aminobenzoic acid (*p*-ABA), and activation of the carboxylic groups of *p*-ABA with EDC/NHS for the immobilization of *p*-aminothiophenol to which gold nanoparticles (AuNPs) were attached. The aptamers were further immobilized on the AuNPs via the interaction of thiol and gold. After blocking the aptamer-free spaces of the AuNPs with mercaptohexanol, the impedimetric measurements were carried out after protein incubation for 60 min, leading to an LOD of 1.6 pg mL^−1^ in buffer [57]. He et al. also reported an aptasensor based on rationally designed peptide aptamers through localized surface plasmon resonance imaging for real-time and label-free detection of IL-6 in serum samples from patients with COVID-19. For the sensor development, a glass substrate was initially patterned with gold nanorods conjugated with thiolated aptamers, and the chip was further mounted under an optical dark-field microscope coupled with an electron-multiplying charge-coupled device. Using this specific biosensing platform, the obtained LOD was reported as 4.6 pg mL^−1^ in serum samples [58].

Electrochemically synthesized MIPs have also been reported for the detection of IL-6 [11, 30, 31, 33, 59]. Yaman et al. developed an impedimetric electrochemical MIP-based sensor through whole-protein imprinting of polydopamine. To enhance signal-to-noise ratios and increase the effective surface area, screen-printed gold electrodes were functionalized with peptide nanotubes, resulting in a theoretical LOD of 0.25 pg mL^−1^ [30]. In addition, Goncalves et al. prepared a point-of-care sensing platform based on whole-protein imprinting of IL-6 on carbon screen-printed electrodes (C-SPEs). Initially, IL-6 was mixed with a solution of pyrrole and carboxylated pyrrole for 30 min to actuate the pre-complexation of the template and functional monomers through hydrogen bonds. Secondly, after their pretreatment, C-SPEs were incubated with the pre-complexation solution, and the monomers were electropolymerized using cyclic voltammetry (CV). The template was removed through chemical cleavage by incubating the electrodes with an acidic solution for 3 h. Finally, different concentrations of IL-6 solution in buffer and serum were incubated on MIP-functionalized C-SPEs for 30 min prior to detection. The study reported different behavior of impedance spectroscopy in buffer and serum, with an LOD lower than 0.1 pg mL^−1^ in buffer and 0.02 pg mL^−1^ in serum samples [11]. Similarly, Ting et al. developed a MIP-based electrochemical sensor through whole-protein imprinting on modified C-SPEs using poly(*o*-phenylenediamine) as the functional monomer. To enhance the sensor performance, several modifications were made to the C-SPEs, including oxygen plasma treatment of C-SPEs prior to AuNP deposition on the surface, immersion of (3-aminopropyl)triethoxysilane, and finally, glutaraldehyde grafting. After modifying the C-SPEs, they were incubated with a pre-complexation solution of the IL-6 with the functional monomer, and the electropolymerization was conducted using CV. The template was removed after incubation of the polymer with salt solution for 2 h. For the detection, IL-6 solutions were incubated with the MIP-functionalized C-SPEs for 15 min, leading to an LOD of 1.74 pg mL^−1^ in buffer [33].

Compared to electropolymerized MIPs, core–shell MIPs provide the opportunity to combine the properties of a functional core with the binding capabilities of MIPs, leading to multifunctionality. In the case of IL-6, there is only one core–shell MIP reported in the literature [12], where the whole protein was imprinted on the surface of protease−copper phosphate hybrid nanoflowers to enrich and further degrade IL-6 into fragments through the biological enzyme characteristics of the core material. The applicability of the particles was tested by measuring the concentration of IL-6 in solution after incubation with the MIPs for a minimum of 24 h. The results showed that MIPs effectively hydrolyzed IL-6 after incubation, and the hydrolysis was higher when incubated for a longer time, whereas the NIPs did not show any hydrolyzing activity. The authors further investigated the catalytic activity of their particles in vitro and in vivo*,* showing downregulation of IL-6 in mice with reduced inflammatory response [12].

Although no core–shell MIPs have been reported for sensing of IL-6, there are reports for the detection of other biomarkers related to infectious complications [60, 61]. Piloto et al. prepared core–shell MIPs through whole-molecule imprinting of acrylamide-based functional monomers on quantum dots (QDs) for the detection of interleukin 2 (IL-2), which is another cytokine from the interleukin family, using fluorescent sensors. The optimization steps indicated that imprinting the protein on the surface of the QDs through surface imprinting resulted in more sensitive detection than bulk imprinting. Prior to detection, the particles were incubated with IL-2 for 30 min, which led to fluorescence quenching by increasing the protein concentration, while the response of QD-NIPs was random signals. The developed sensor assay showed a theoretical LOD of 5.91 fg mL^−1^ in 1000-fold diluted serum [60]. In another work, Xu et al. used an allyl-based deep eutectic solvent as the functional monomer to synthesize the MIP shell through whole-protein imprinting on silica-coated magnetic nanoparticles for the detection of lysozyme, an antimicrobial enzyme that plays essential roles in the immune system. The functionality of the particles was investigated via adsorption experiments, in which MMIPs were incubated with protein solution for 24 h under shaking, and they were subsequently collected using a magnet to determine the protein concentration of the supernatant with a UV–Vis spectrometer. In this study, the maximum binding capacity of the MMIPs was reported as 108 mg g^−1^, with an LOD of 12.8 $\mu$ g mL^−1^ and an IF of 2.28 [61].

Our work shows competitive sensitivity even in complex media such as human serum, with a significantly lower LOD than those of immunosensors or aptasensors (Table S2). The obtained affinity in this work is comparable to that of antibody–antigen, while employing cost-effective recognition elements instead of expensive antibodies or aptamers. Furthermore, the proposed sensing platform requires minimum surface modification steps, less sophisticated operation tools, and lower detection times. Additionally, by employing epitope imprinting instead of whole-protein imprinting, we were able to reduce the synthesis costs and broaden the spectrum of synthesis parameters, since epitopes are more stable than proteins in MIP design. Lastly, the MMIPs proposed in this work provide multifunctionality, which can be harvested across various sensing platforms, and applicability in in vitro/in vivo investigations due to their nanoparticle morphological properties.

## Conclusions

In this study, we successfully developed magnetic molecularly imprinted core–shell structures within optimized fabrication conditions. The optimal condition for peptide immobilization was found to be a peptide concentration of 0.1 mM in PBS, whereas the optimal T:FM:CL ratio was determined to be 1:20:20, resulting in sufficient cross-linking to obtain a stable polymeric layer. Moreover, multiple characterization tools confirmed the formation and preservation of the polymer shell around the MNPs after polymerization and TR, respectively. The MMIPs-SPE sensing platform could detect the target protein IL-6 in only 15 min of protein incubation with high sensitivity (LOD of 0.00038 pM) and affinity (*K*_D_ of 0.25 pM) in buffer. This increased sensitivity was also observed in human serum with an experimentally determined detection limit of 0.38 pM. The selectivity studies revealed that the response from MMIPs was more than four times that observed from MNIPs. Also, the specificity studies showed that the highest signal was associated with IL-6 cytokine.

By providing a comprehensive protocol outline, this study paves the way for designing and developing multifunctional MMIP core–shell structures, which can be further employed for early detection of pathologies using magnetoelectric, fluorescence, and electrochemical sensor readouts, while it expands the potential for prognosis and imaging applications both in vitro and in vivo.

## Supplementary Information

Below is the link to the electronic supplementary material.

ESM 1 (DOCX 2.9 MB)
