# Supplementary material for: Synthesis and characterization of core–shell magnetic molecularly imprinted polymer nanocomposites for the detection of interleukin-6
Source: Anal Bioanal Chem. 2024 Oct 16;416(28):6237–57. doi: 10.1007/s00216-024-05536-x (PMC11541377; doi:10.1007/s00216-024-05536-x)
Supplement: Supplementary file 1 — (DOCX 2.9 MB) [file 216_2024_5536_MOESM1_ESM.docx]

**Analytical and Bioanalytical Chemistry**

**SUPPORTING INFORMATION**

**Synthesis and characterization of core-shell magnetic molecularly imprinted polymer nanocomposites for the detection of Interleukin-6**

Rahil Radfar^1^, Eda Akin^1^, Ekin Sehit^1^, Nastasia Sanda Moldovean-Cioroianu^1^, Niklas Wolff^2^, Rodrigue Marquant^3^, Karsten Haupt^3,4^, Lorenz Kienle^2,5^, Zeynep Altintas^1,5^*

^1^ Bioinspired Materials and Biosensor Technologies, Institute of Materials Science, Faculty of Engineering, Christian-Albrechts-Universität zu Kiel, Germany

^2^ Real Structure and Synthesis, Institute of Materials Science, Faculty of Engineering, Christian-Albrechts-Universität zu Kiel, Germany

^3^ CNRS Enzyme and Cell Engineering Laboratory, Universite de Technologie de Compiègne, France

^4^ Institut Universitaire de France, France

^5^ Kiel Nano, Surface and Interface Science (KiNSIS), Kiel University, Germany

*Corresponding author: zeynep.altintas@tf.uni-kiel.de

**Table of contents**

1. **Mathematical approach for determining the saturation concentrations**……………………..………………..2

**Fig. S1** Mass spectroscopy and high-performance liquid chromatography analyses of the peptide..…………………….…….….3

**Fig. S2** PEPFOLD prediction of amino acid residues of the peptide…………………………………………………….…….…..4

**Fig. S3** Projection areas for peptide and citric acid, and UV-Vis spectra of MNPs and EDC/NHS in aqueous solutions…….…..4

**Fig. S4** Experimental analysis for finding the optimum concentration of peptide, and the reaction media……………………..5

**Fig. S5** Sample treated with MeOH:AAc (100%), and the FTIR spectra of different stages of the synthesis…………………..5

**Fig. S6** FTIR, DLS, and ELS characterizations of different concentrations of ${TR}_{MeOH:AAc}$. ………………………………….6

**Fig. S7** Emission and excitation spectra if the sample treated with ${TR}_{MeOH:AAc/50\%}$………………………………………....7

**Fig. S8** Possible formation of polymer shells on the surface of the MNPs…………………………………………….7

**Fig. S9** Isoelectric point of peptide calculated using MarvinSketch software……………………………………………...8

**Fig. S10** The DLS and ELS results of different TR solutions on samples with T:FM:CL=1:20:20………………………..…..9

**Fig. S11** FTIR and Fluorescent spectroscopy of different TR solutions on samples with T:FM:CL=1:20:20........................9

**Table S1** Experimental parameters of SWV measurement for epitope rebinding studies…….………………………10

**Table S2** Comparison of this work with other detection assays………………………………………………………10

1. **Mathematical approach for determining the saturation concentration of EDC/NHS, and the peptide**

$$A_{MNP}=4\pi r^{2}=8000 {nm}^{2}$$

$$Max. projection area of citric acid (CT) \approx0.5819 {nm}^{2} \to\frac{A_{MNP}}{A_{CT}}=13500$$

$$each CT has 3 -COOH group$$

$$\to No. -COOH on each MNP=40500$$

$$\leftrightarrow\boldsymbol{1 mol of MNP \propto40000 mol of EDC}$$

On the other hand,

$$Max. projection area of peptide \approx2.3217 {nm}^{2}$$

$$\to\frac{A_{MNP}}{A_{pep(\max)}}=3445$$

$$Min. projection area of peptide \approx1.4611 {nm}^{2}$$

$$\to\frac{A_{MNP}}{A_{pep(min)}}=5475$$

$$\leftrightarrow\boldsymbol{1 mol of MNP \propto in average 4000 mol of peptide}$$

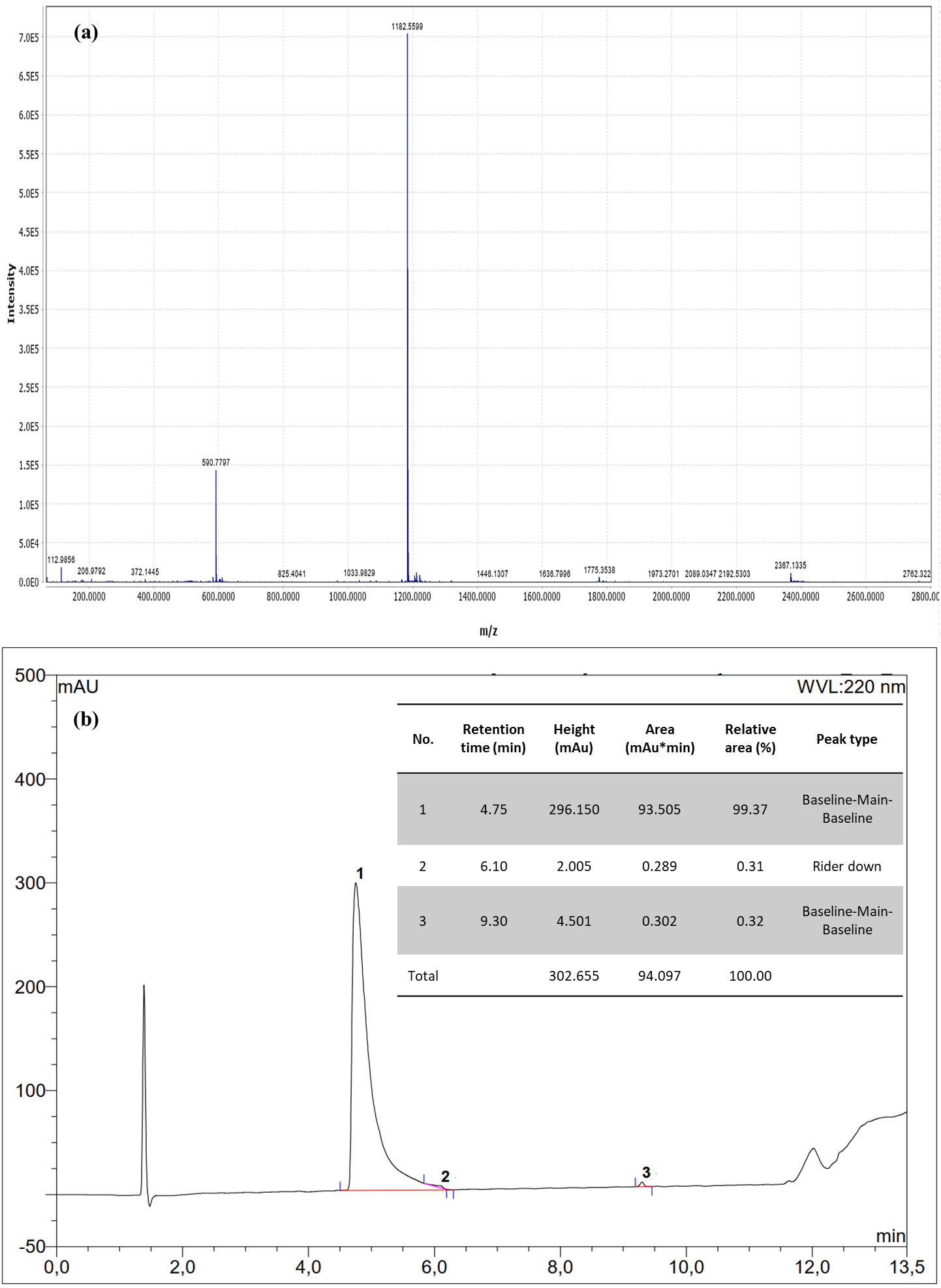


**Fig. S1** (a) Mass spectroscopy (MS) and (b) high-performance liquid chromatography (HPLC) analyses of the peptide. The HPLC method involved a gradient elution from 5% to 30% acetonitrile over 10 minutes, with a UV-VIS detection at 220 nm and a bandwidth of 4 nm. It was performed on a C18 column with 3-micron particle size and dimensions of 4.6 mm x 100 mm


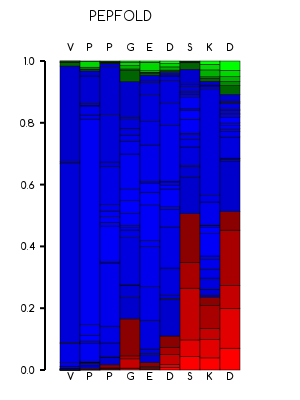


**Fig. S2** PEPFOLD prediction of amino acid residues of the peptide with green showing extended structure, blue denoting to a coil, and red to a helical secondary structure

**
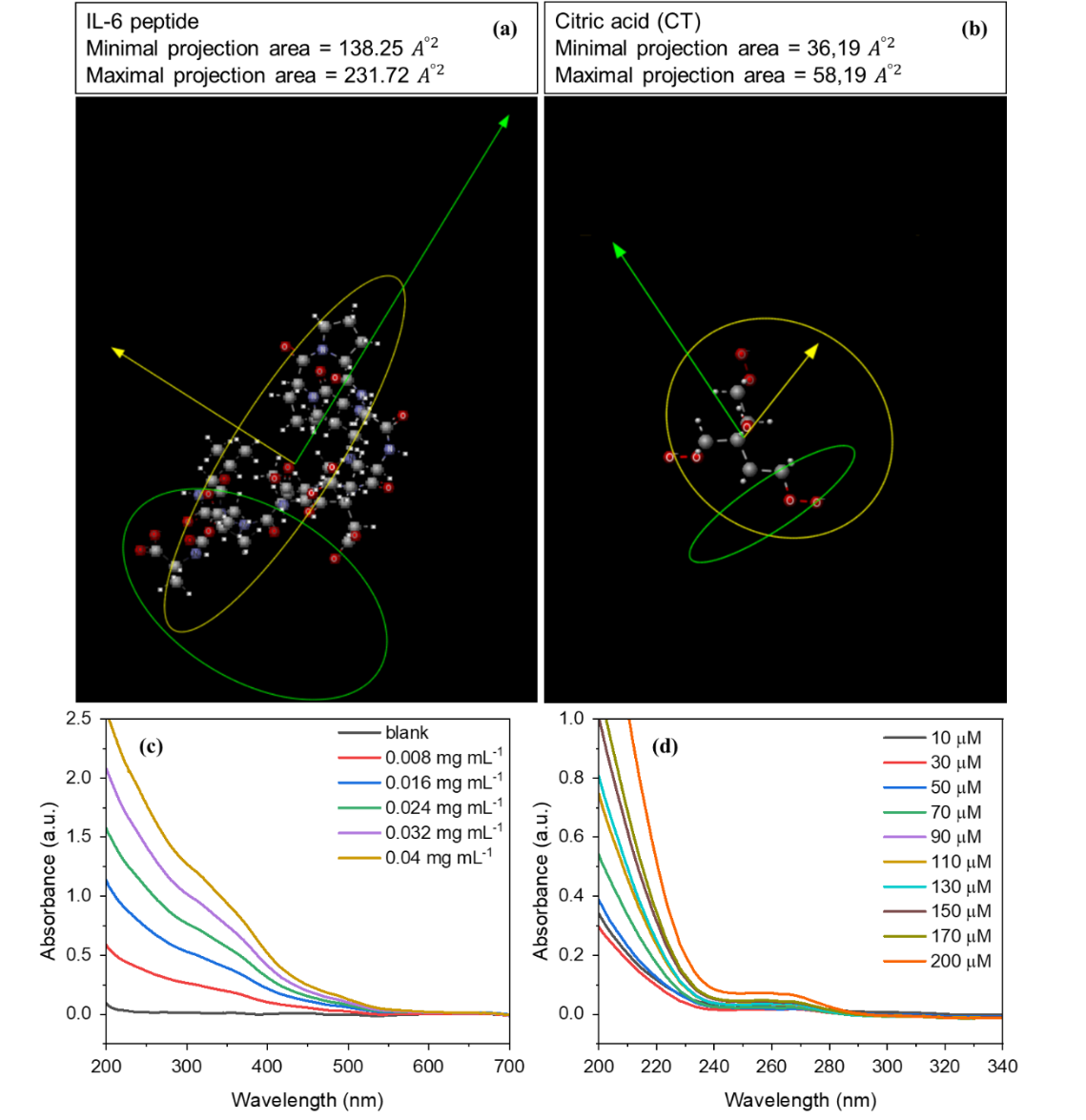
**

**Fig. S3** Maximal and minimal projection areas of (a) the peptide, and (b) citric acid obtained using MarvinSketch software, and UV-Vis spectra of increasing concentrations of (c) MNPs, and (d) EDC/NHS in aqueous solutions. It is clear from the images that the hydrolysis peak of EDC/NHS at 260 nm would be overlaid by the very high peaks of the MNPs in that region, resulting in a not-reliable titration analysis


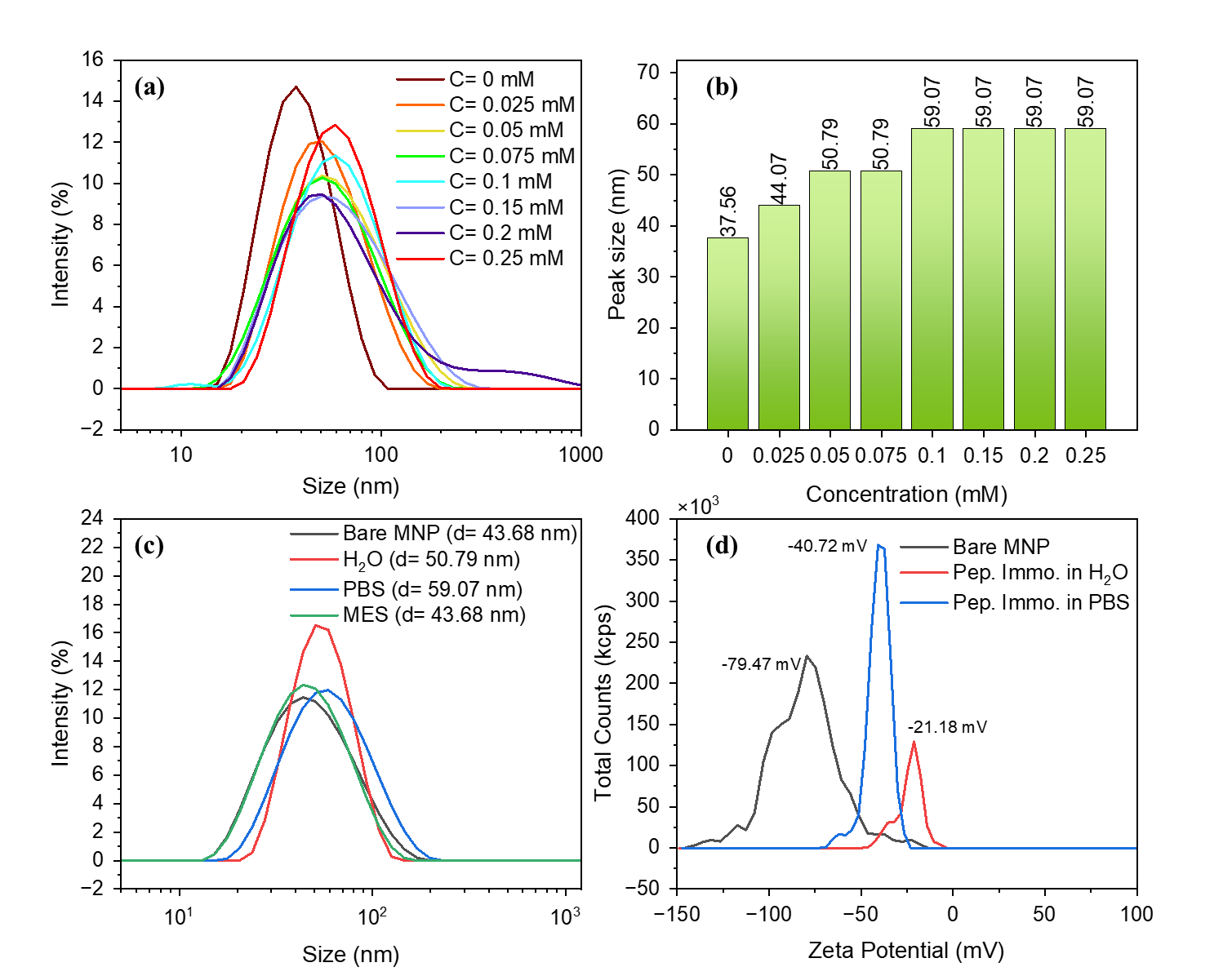


**Fig. S4** Experimental analysis for finding the optimum concentration of peptide, and the reaction media. (a) The hydrodynamic size distribution of MNPs after functionalization with different peptide concentrations, (b) the corresponding bar chart comparing the peak sizes of particles, (c) the DLS and (d) ELS analyses of peptide-immobilized MNPs in different media of reactions including water, PBS, and MES

**
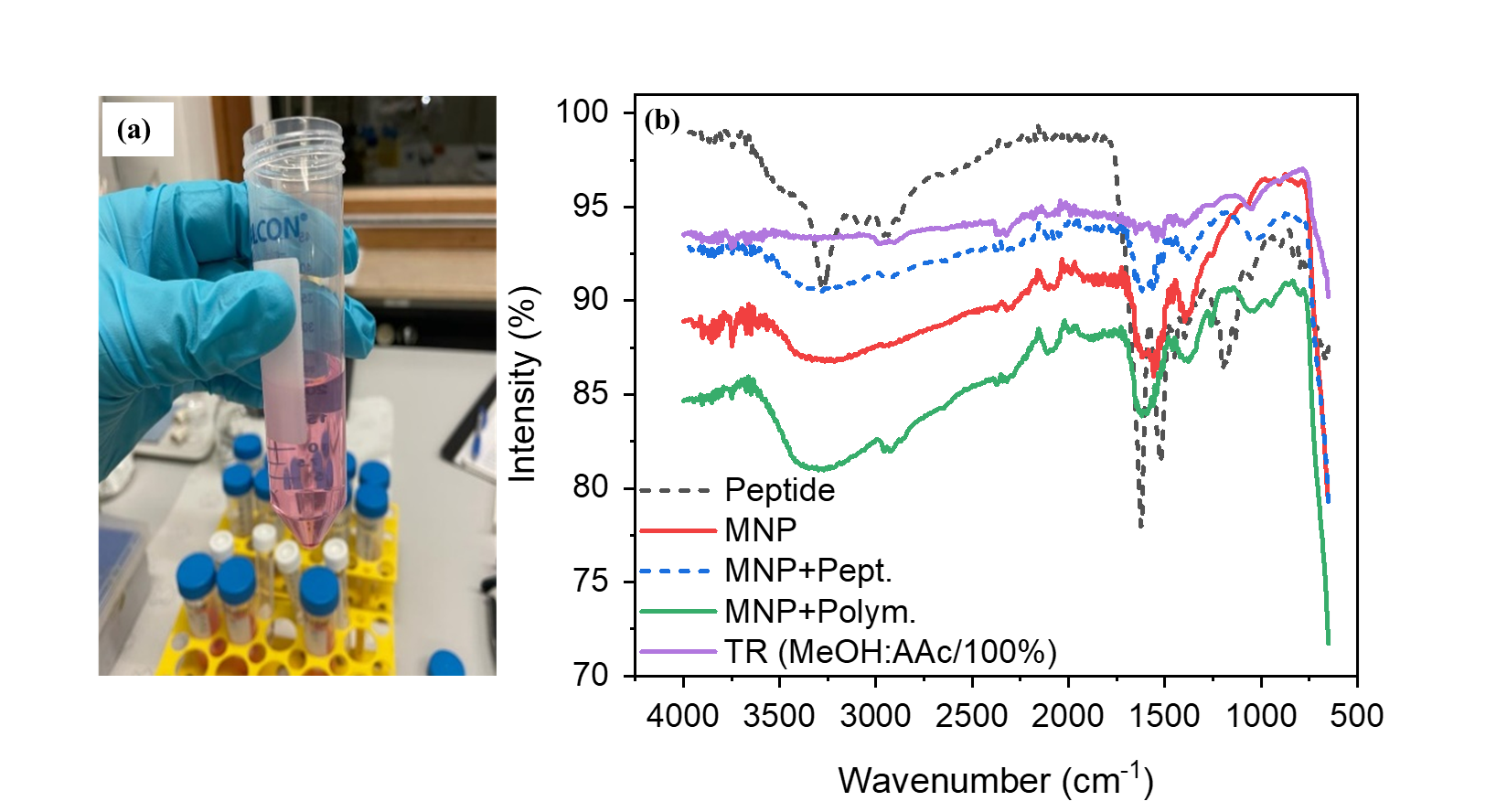
**

**Fig. S5** (a) An image of the sample treated with MeOH:AAc (100%) as the TR after centrifugation, and (b) the FTIR spectra of samples at different stages of the synthesis


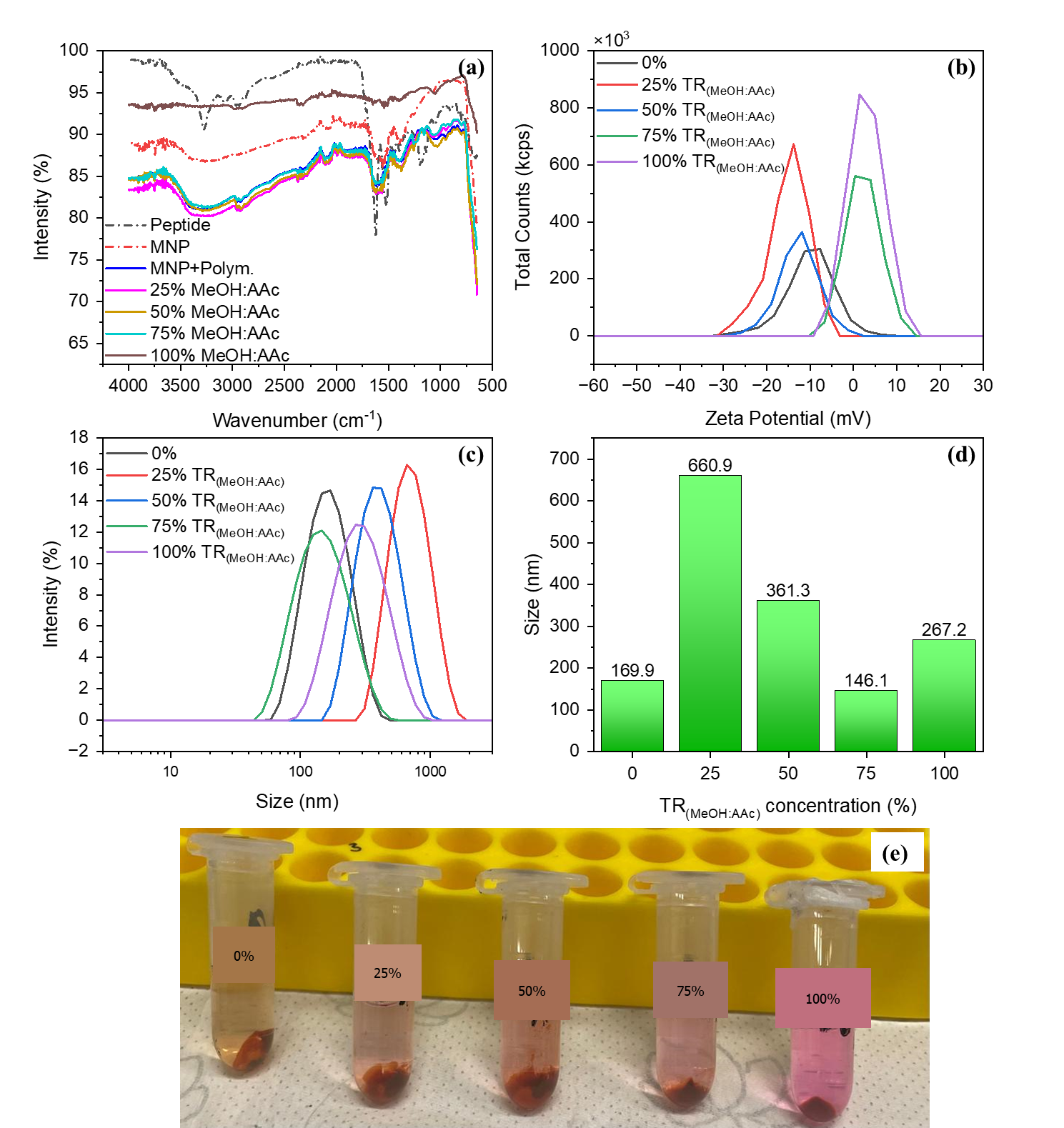


**Fig.** **S6** (a) FTIR, (b) ELS, and (c) DLS characterizations of the samples treated with different concentrations of ${TR}_{MeOH:AAc}$. (d) The bar chart depicts the peak hydrodynamic size of the samples, and (e) the image shows different samples after centrifugation step upon treatment with ${TR}_{MeOH:AAc}$


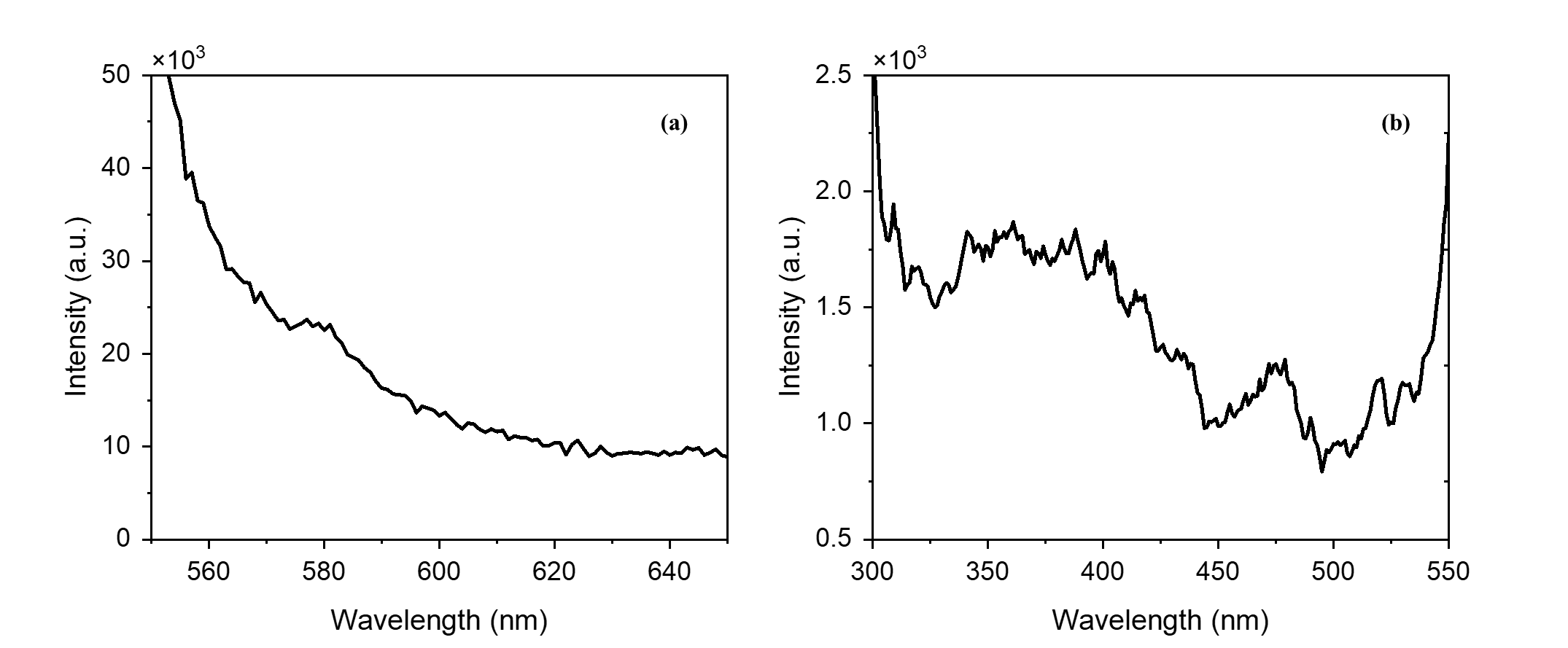


**Fig. S7** (a) Emission and (b) excitation spectra of the sample treated with ${TR}_{MeOH:AAc/50\%}$. The fluorescent emission spectrum of the particles, excited at 530 nm, showed a drastically small peak at around 570 nm, while excitation spectrum, with an emission wavelength at 570 nm, did not match that of RhB monomer with an excitation peak at 540 nm


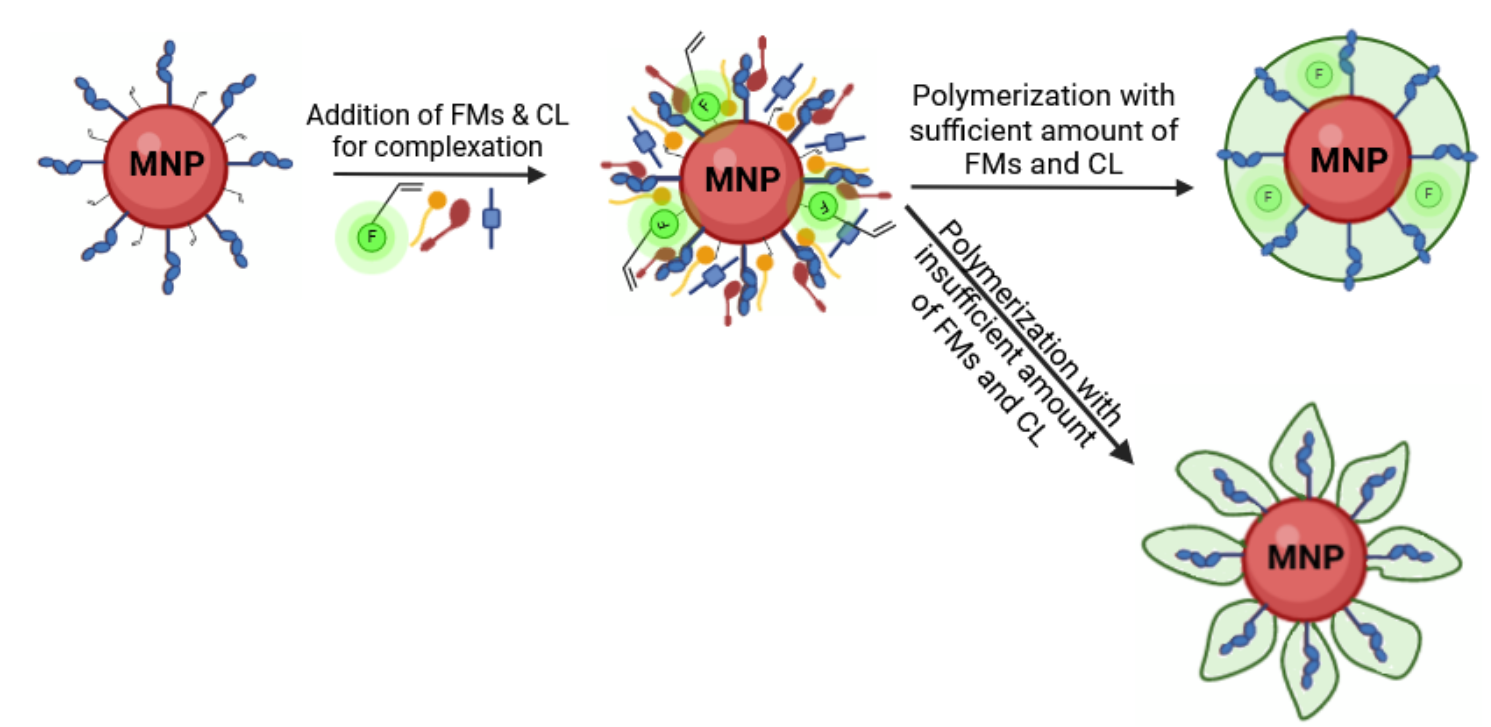


**Fig.** **S8** Possible formation of polymer shells on the surface of the MNPs in the case of having sufficient or insufficient FM and CL agents


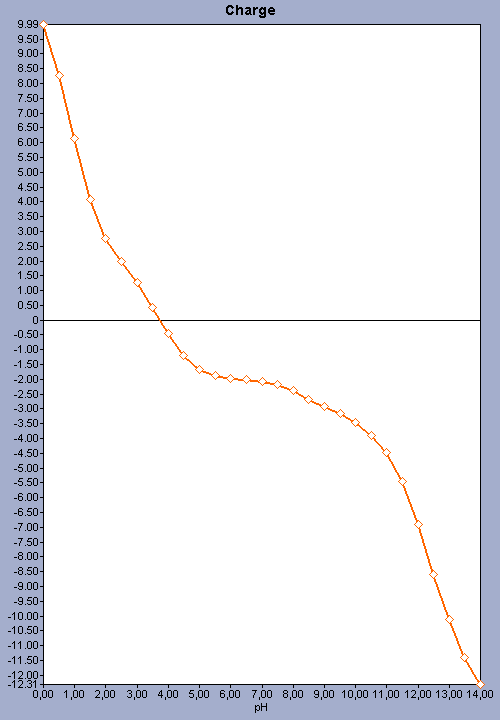


**Fig. S9** Isoelectric point of peptide calculated using MarvinSketch software


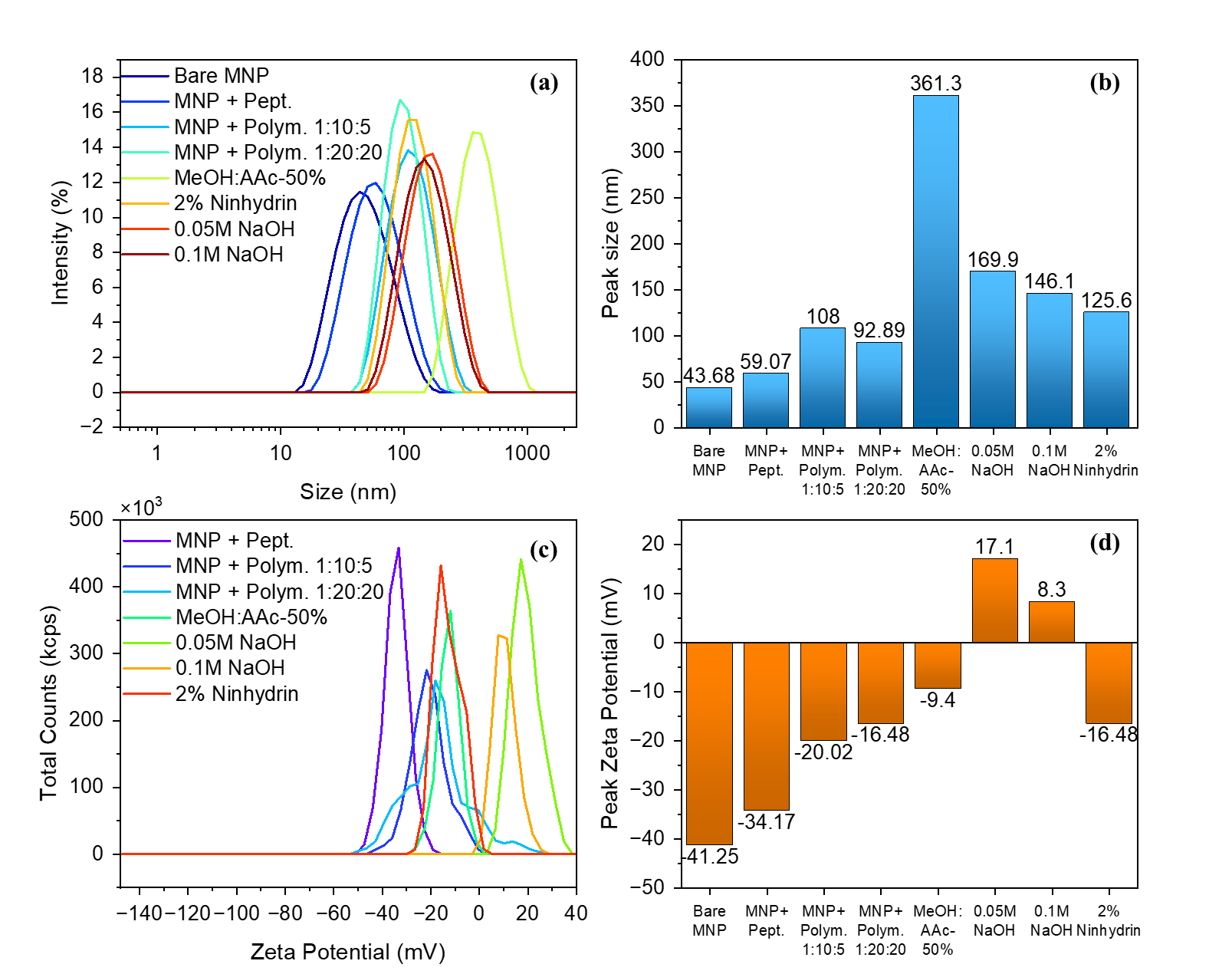


**Fig. S10** (a) The DLS results, and (b) their corresponding bar chart, along with the (c) ELS results, and (d) their corresponding bar chart of the samples with the new formulation (T:FM:CL=1:20:20) treated with different TR solutions. The sample polymerized with T:FM:CL=1:20:20 possessed smaller hydrodynamic size in comparison to 1:10:5 confirming higher amount of crosslinking. The treatment with ${TR}_{MeOH:AAc/50\%}$ showed a boost in the hydrodynamic size of particles indicating that this solution was not a good solvent and led to large agglomerates, whereas the size difference in samples treated with NaOH and ninhydrin was lower. The ZP with most of the TR solutions was negative except from the ones treated with NaOH with a positive ZP


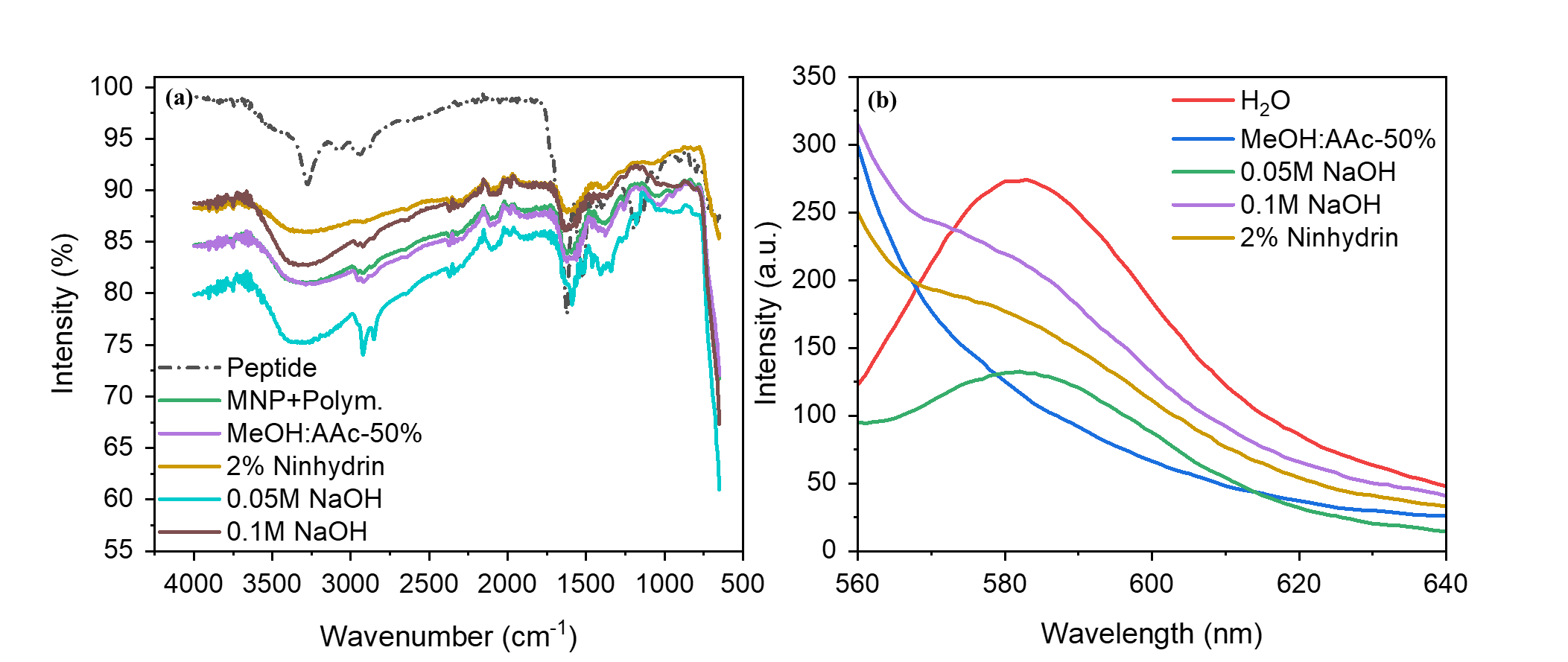


**Fig. S11** The (a) FTIR, and (b) fluorescent emission spectroscopy results of the samples with the new formulation (T:FM:CL=1:20:20) treated with different TR solutions

**Table S1** Experimental parameters of SWV measurement for epitope rebinding studies.

| **SWV method** |
| --- |
| Current Range |
| 1µA – 1 mA |
| Pretreatment settings |
| E condition = 0 V |
| t condition = 0 s |
| E deposition = 0 V |
| t deposition = 0 s |
| Measurement settings |
| t equilibration = 1 s |
| E begin = - 0.3 V |
| E end = 0.9 V |
| E step = 0.003 V |
| Amplitude = 0.05 V |
| Frequency = 10 Hz |

**Table S2** Comparison of this work with other detection assays

| Sensor | Bioreceptor | Assay type | Detection time | Affinity | LOD | IF | Ref. |
| --- | --- | --- | --- | --- | --- | --- | --- |
| Electrochemical immunosensor | Capture antibody conjugated magnetic nanobeads | Sandwich | overnight | ... | 0.42 pg mL^-1^ in buffer | ... | [55] |
| Electrochemical immunosensor | Capture antibodies | Direct | 90 minutes | ... | 5.1 pg mL^-1^ in buffer | ... | [56] |
| Electrochemical aptasensor | Aptamers | Direct | 60 minutes | ... | 1.6 pg mL^-1^ in buffer | ... | [57] |
| Localized surface plasmon resonance imaging based aptasensor | Peptide aptamers | Direct | Real-time | ... | 4.6 pg mL^-1^ in serum | ... | [58] |
| Electrochemical MIP-based sensor | Whole-protein imprinted electroMIPs | Direct | ... | ... | theoretical value of 0.25 pg mL^-1^ | … | [30] |
| Electrochemical MIP-based sensor | Whole-protein imprinted electroMIPs | Direct | 30 minutes | ... | 0.1 pg mL^-1^ in buffer, and 0.02 pg mL^-1^ in serum samples | ... | [11] |
| Electrochemical MIP-based sensor | Whole-protein imprinted electroMIPs | Direct | 15 minutes | ... | 1.74 pg mL^-1^ in buffer | ... | [33] |
| Fluorescent sensors | IL-2 whole- protein imprinted core-shell MIPs on quantum dots | Direct | 30 minutes | ... | 5.91 fg mL^-1^ in 1000 times diluted serum | ... | [60] |
| Adsorption based optical sensors | Lysozyme whole- molecule imprinted core-shell MIPs on silica-coated magnetic nanoparticles | Direct | 24 hours | ... | 12.8 $\mu$g mL^-1^ in buffer | 2.28 | [61] |
| Electrochemical | Magnetic molecularly imprinted polymers | Direct | 15 minutes | 0.25 pM in buffer and 1.6 pM in serum | 0.00038 pM, i.e., 0.01 pg mL^-1^, in buffer, and 0.38 pM, i.e., 10 pg mL^-1^, in serum | 4.28 | This work |
